# The associations between healthful and unhealthful plant-based dietary patterns and all-cause mortality among older Chinese adults with data-adapted early-stage cardiovascular-kidney-metabolic syndrome (stages 0–2): a prospective cohort study from the CLHLS

**DOI:** 10.3389/fnut.2026.1915953

**Published:** 2026-10-16

**Authors:** Shipeng Zhang, Enjie Tang, Yanjie Jiang, Kun Zhu, Xiulian Zhuang, Hanyu Wang, Xingyi He, Yan Lu, Qinxiu Zhang, Xueying Li, Jiaqing Song

**Affiliations:** 1 Chengdu University of Traditional Chinese Medicine, Chengdu, China; 2 Guangzhou University of Chinese Medicine, Guangzhou, China; 3 Department of Neurology, The 925th Hospital of PLA Joint Logistics Support Force, Guiyang, China; 4 Nanjing University of Chinese Medicine, Nanjing, China; 5 Hospital of Chengdu University of Traditional Chinese Medicine, Chengdu, China; 6 Department of Cardiology, The 982th Hospital of PLA Joint Logistics Support Force, Tangshan, Hebei, China

**Keywords:** all-cause mortality, cardiovascular-kidney-metabolic, HPDI, plant-based dietary patterns, uPDI

## Abstract

**Aims:**

To examine the associations of the overall plant-based diet index (PDI), healthful plant-based diet index (hPDI), and unhealthful plant-based diet index (uPDI) with all-cause mortality in older Chinese adults with data-adapted early-stage cardiovascular-kidney-metabolic (CKM) syndrome (stages 0–2).

**Methods and results:**

We analyzed data from 5,872 participants aged ≥65 years from the 2008 wave of the China Longitudinal Healthy Longevity Survey (CLHLS) with data-adapted CKM stages 0–2 and no prevalent cardiovascular disease. Dietary intake was assessed using a validated food frequency questionnaire. Mortality was ascertained through March 2019. Cox proportional hazards models and restricted cubic spline analyses were used. During a median follow-up of 108 months, 2,870 deaths occurred. In multivariable-adjusted models, each 1-point increase in hPDI was associated with a 1.1% lower mortality risk (HR = 0.989, 95% CI: 0.982–0.995; *p* < 0.001), whereas each 1-point increase in uPDI was associated with a 1.2% higher mortality risk (HR = 1.012, 95% CI: 1.006–1.019; *p* < 0.001). Compared with the lowest quartile, the highest hPDI quartile had a 13.2% lower mortality risk (HR = 0.868, 95% CI: 0.783–0.961), and the highest uPDI quartile had a 19.2% higher risk (HR = 1.192, 95% CI: 1.060–1.340). Stratified estimates suggested numerically stronger associations in CKM stages 1–2 than in stage 0; however, formal interaction tests did not reach statistical significance (*p* = 0.062 for hPDI and *p* = 0.824 for uPDI), indicating limited evidence of effect modification.

**Conclusion:**

Higher adherence to a healthful plant-based diet was associated with lower all-cause mortality, whereas higher adherence to an unhealthful plant-based diet was associated with higher mortality among older Chinese adults with data-adapted early-stage CKM syndrome. The overall PDI was not independently associated with mortality after adjustment, underscoring that plant-based dietary quality, rather than plant-based eating per se, is the relevant exposure.

## Graphical abstract

**Figure:**
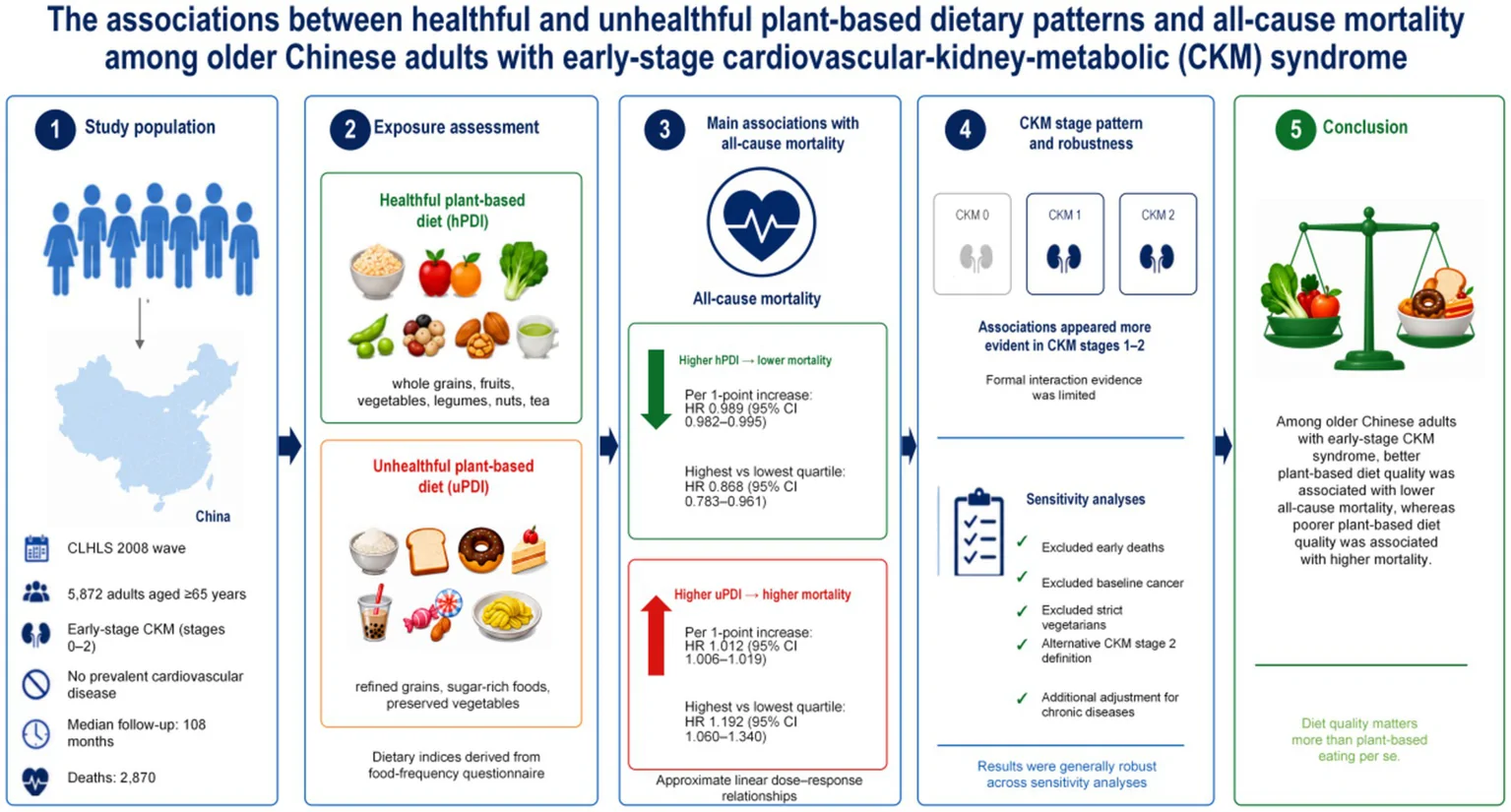

## Highlights

*What is already known*

- Cardiovascular-kidney-metabolic (CKM) syndrome provides a framework for identifying individuals with early cardiometabolic and kidney-related risk before overt cardiovascular disease develops.
- Plant-based dietary patterns differ in quality: healthful plant foods have been associated with lower mortality risk, whereas unhealthful plant foods rich in refined grains, sugar, or preserved products may confer less benefit.
- Evidence on plant-based diet quality and mortality among older Chinese adults with early-stage CKM syndrome remains limited.

*What this study adds*

- In 5,872 older Chinese adults with data-adapted CKM stages 0–2 from the CLHLS, higher hPDI was associated with lower all-cause mortality, whereas higher uPDI was associated with higher mortality during long-term follow-up.
- The overall PDI was inversely associated with mortality in unadjusted analyses but not after multivariable adjustment, supporting the importance of dietary quality over plant-based eating per se.
- Stratified estimates were numerically stronger among participants with CKM stages 1–2 than among those with CKM stage 0; however, formal interaction tests were not statistically significant, providing limited evidence for effect modification by CKM stage.
- The findings were robust across multiple sensitivity analyses and support focusing on the quality of plant-based foods rather than plant-based eating per se in early CKM risk management.

## 1 Introduction

Cardiovascular-kidney-metabolic (CKM) syndrome, characterized by the confluence of early-stage cardiovascular disease, chronic kidney disease, and metabolic dysregulation, poses a substantial global health burden (1, 2). Recently, the American Heart Association (AHA) emphasized the importance of recognizing CKM syndrome as a systemic condition, given its significant implications for cardiovascular disease (CVD) prevention and management (3). In primary care, CKM stages can identify high-risk individuals earlier than conventional risk scores by incorporating renal and subclinical cardiovascular parameters, enabling timely intensification of therapies with proven cardio-renal-metabolic benefit. Notably, even during the preclinical stages (CKM stages 0–2), individuals exhibit a progressive increase in cardiovascular and all-cause mortality, underscoring a critical window for early preventive interventions targeting modifiable risk factors (4, 5).

Among potential interventions, dietary patterns—particularly plant-based diets—have emerged as critical modifiable factors. Contemporary research differentiates healthful plant-based diet index (hPDI), characterized by a high intake of fruits, vegetables, legumes, whole grains, and nuts, from unhealthful plant-based diet index (uPDI), which are dominated by refined grains, sugar-sweetened foods, and other low-nutrient plant products (6). Prospective studies consistently demonstrate that higher hPDI adherence is associated with lower all-cause and cardiovascular mortality, whereas higher uPDI intake is associated with a higher risk (7, 8). Consistent findings have been reported for overall plant-based eating in other populations: greater adherence to a plant-based diet was associated with a lower risk of incident cardiovascular disease, cardiovascular disease mortality, and all-cause mortality among middle-aged US adults (9), and improvements in plant-based diet quality over time were associated with lower total and cause-specific mortality (10). Likewise, higher plant protein intake, particularly when replacing animal protein, has been associated with lower mortality risk (11). Beyond individual diseases, international dietary frameworks such as the EAT–Lancet Commission advocate plant-forward dietary patterns for both human health and environmental sustainability (12). For instance, older adults with metabolic syndrome or CKM traits in the highest hPDI quartile exhibited an up to 60% lower cardiovascular mortality rate, while those in the highest uPDI quartile demonstrated a 59–75% higher mortality risk (13). Mechanistically, these protective effects are mediated through the attenuation of insulin resistance, systemic inflammation, and oxidative stress, alongside the enhancement of endothelial function—all of which are central to CKM pathophysiology (5, 14). The 2023 AHA advisory further emphasizes that systemic inflammation, oxidative stress, insulin resistance, and declining kidney function collectively drive the transition from early to advanced CKM stages, providing a mechanistic rationale for dietary and lifestyle interventions targeting these pathways (15).

Despite this growing evidence, the impact of plant-based diet quality on mortality among older Chinese adults with early-stage CKM (stages 0–2) remains largely underexplored. Although the CLHLS has enabled landmark analyses of plant-based dietary patterns and mortality among Chinese older adults (16), whether plant-based diet quality, as captured by the hPDI, uPDI, and overall PDI, relates to mortality specifically among individuals with early-stage CKM has not been examined. The Chinese Longitudinal Healthy Longevity Survey (CLHLS) provides a unique opportunity to investigate this association over a 11-year period (2008–2019), leveraging comprehensive dietary assessments and longitudinal mortality follow-up data. By applying validated hPDI and uPDI indices, we aim to quantitatively capture both protective and deleterious plant-based dietary patterns, thereby facilitating a more precise evaluation of dietary prevention strategies in early-stage CKM.

Therefore, this study aimed to examine the associations of the overall PDI, hPDI, and uPDI with all-cause mortality among older adults with CKM stages 0–2, focusing on whether adherence to a healthful plant-based diet confers survival benefits, and whether unhealthful plant-based consumption is associated with a higher mortality risk. Elucidating these associations is expected to inform early dietary interventions and precision nutrition strategies, ultimately contributing to mortality reduction and healthier aging within the older Chinese population.

## 2 Methods

### 2.1 Study design and population

This prospective cohort study used data from the CLHLS, a nationally representative survey covering 23 provinces of China. Details of the CLHLS have been published elsewhere (7). The present analysis included participants aged 65 years or older from the 2008 wave, with follow-up until 2019. After excluding individuals with missing data on dietary intake, covariates, or survival status, 5,872 participants with CKM stages 0–2 were included in the final analytical sample (Figure 1).

**Figure 1:**
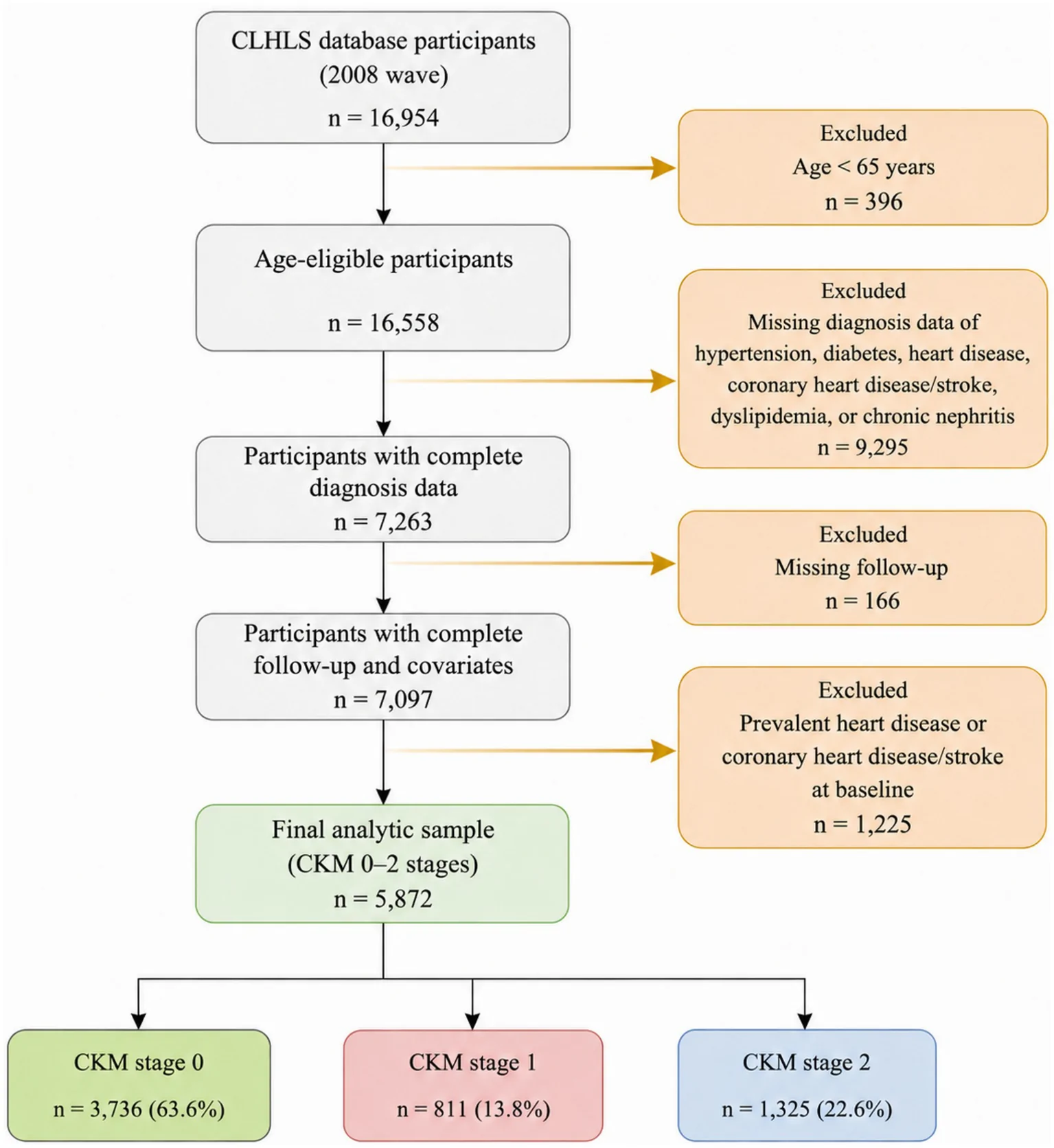
Flowchart of participant selection from the CLHLS 2008-2018 cohort.

### 2.2 Assessment of plant-based diet indices

Dietary intake was assessed using a validated food frequency questionnaire administered during face-to-face interviews. In accordance with previously established methods (7, 16), two plant-based diet indices were constructed: hPDI and an uPDI. Briefly, 16 food groups were classified into three categories: (i) healthful plant foods (e.g., whole grains, fresh fruits, fresh vegetables, legumes, nuts, tea); (ii) unhealthful plant foods (e.g., refined grains, preserved vegetables, sugar); and (iii) animal foods (e.g., meat, fish, eggs, dairy). Each food group was scored according to frequency of consumption, ranging from 1 (least frequent) to 5 (most frequent). For the hPDI, positive scores were assigned to healthful plant foods, whereas unhealthful plant foods and animal foods were reverse-scored. For the uPDI, positive scores were assigned to unhealthful plant foods, while healthful plant foods and animal foods were reverse-scored. The total score for each index ranged from 16 to 80, with higher hPDI and lower uPDI indicating a healthier plant-based dietary pattern. Detailed scoring criteria are provided in Supplementary Table S3.

In addition, the overall plant-based diet index (PDI) was constructed following established methods (6): all plant food groups received positive scores and all animal food groups received reverse scores, irrespective of food healthfulness. The PDI therefore reflects the overall degree of plant-based eating without distinguishing dietary quality; its inclusion facilitates comparison with previous literature using all three indices. Detailed scoring criteria are provided in Supplementary Table S3.

### 2.3 CKM syndrome staging (0–2 stages)

This study adopted the CKM syndrome staging recommended by the 2023 AHA President’s Recommendations, with adaptive adjustments based on CLHLS data availability (1). Because the database lacked continuous measures of renal function (e.g., serum creatinine, urinary albumin) and blood lipids (e.g., triglycerides, HDL), the staging relied on self-reported physician diagnoses and participants’ physical measurements.

#### 2.3.1 CKM 0 stage

Body Mass Index (BMI) < 24.0 kg/m^2^ (standard for the Chinese population), waist circumference for men < 90 cm and for women < 85 cm, and no hypertension, diabetes, dyslipidemia, or chronic nephritis (all based on doctor’s diagnosis), no history of cardiovascular or cerebrovascular diseases. This stage reflects a data-adapted operational definition based on available anthropometric measurements and self-reported clinical information and should not be interpreted as biomarker-confirmed absence of cardiometabolic risk.

#### 2.3.2 CKM 1 stage

BMI ≥ 24.0 kg/m^2^ or exceeding the waist circumference limit (for men ≥ 90 cm, for women ≥ 85 cm), but without hypertension, diabetes, dyslipidemia, or chronic nephritis.

#### 2.3.3 CKM 2 stage

Presence of any one or more of the doctor-diagnosed hypertension, diabetes, dyslipidemia, or chronic nephritis, regardless of BMI and waist circumference levels.

Those who self-reported having coronary heart disease, angina pectoris, myocardial infarction, heart failure, or stroke at baseline were excluded as they belong to CKM 4 stage. This data-adapted staging approach was designed to approximate early CKM stages within the constraints of the CLHLS data, while acknowledging the potential for misclassification due to the absence of laboratory-based renal and lipid biomarkers.

Given the absence of laboratory-based renal and lipid biomarkers, we further evaluated the robustness of this data-adapted CKM definition in sensitivity analyses by excluding participants who were classified as CKM stage 2 solely on the basis of physician-diagnosed dyslipidaemia or chronic nephritis. This approach was used to assess whether the main findings were materially influenced by CKM stage 2 assignments that depended entirely on self-reported kidney or lipid-related diagnoses rather than objective laboratory measurements.

### 2.4 Ascertainment of covariates

Baseline covariates were selected *a priori* based on previous CLHLS studies and their potential associations with dietary patterns and mortality. All covariates were obtained from the 2008 baseline survey through face-to-face interviews and physical measurements. The multivariable-adjusted model included demographic factors, socioeconomic characteristics, lifestyle factors, adiposity, and sleep-related variables, including age, sex, ethnicity, marital status, educational attainment, income, occupation, smoking status, drinking status, regular exercise, sleep duration, and sleep quality. Detailed definitions and coding of these covariates were provided in Supplementary Table S1.

Briefly, sleep quality was self-reported on a five-level scale (very good, good, fair/so-so, bad, or very bad) and was modeled as a categorical variable; regular exercise was defined as self-reported current regular exercising (yes/no) (Supplementary Table S1).

Baseline BMI was not included in the primary multivariable model because it was used to define CKM stage 1, while hypertension, diabetes, dyslipidaemia, and chronic nephritis were not included because they were used to define CKM stage 2. These variables were additionally adjusted for in an extended sensitivity model.

### 2.5 Ascertainment of deaths

The CLHLS recorded death information using official death certificates whenever possible; otherwise, data were obtained from close relatives of the participant or the residents committee. Vital status follow-up continued through March 2019. Cause-of-death information was not available in the data extract used for this study; therefore, cause-specific mortality could not be analyzed (16).

### 2.6 Statistical analysis

#### 2.6.1 Study population and exposure assessment

Participants were classified according to baseline CKM stage (0–2) and dietary patterns using the hPDI and uPDI. All three dietary indices (PDI, hPDI, and uPDI) were analyzed as continuous variables and in quartiles. Baseline characteristics across CKM stages were compared using the Kruskal–Wallis test for continuous variables and Pearson’s chi-squared test for categorical variables. Participants with missing key diagnostic variables required for CKM staging or missing vital status were excluded. The baseline characteristics of the 9,295 excluded participants were compared with those of the analytical sample and are presented in Supplementary Table 12. Multiple imputation (m = 5) by chained equations was then applied to covariates with low missingness among the analytic sample (see Supplementary Table S2). Given the low proportion of missingness (maximum 7.1% for household income), five imputed datasets are expected to provide more than 98% relative efficiency compared with an infinite number of imputations, and five imputed datasets were therefore considered sufficient (17, 18).

#### 2.6.2 Primary outcome and regression models

The primary endpoint was all-cause mortality during follow-up. Associations between dietary indices and mortality were estimated using Cox proportional hazards regression models. Two pre-specified models were constructed:

- Model 1 (crude): unadjusted.
- Model 2 (multivariable-adjusted): adjusted for age, sex, ethnicity, marital status, education, smoking, alcohol consumption, household income, physical activity, occupation, sleep duration, and sleep quality.

Proportional hazards assumptions were assessed by inspecting Schoenfeld residuals for each covariate and for the global model. No significant violations were detected (all *p* > 0.05), indicating that the proportional hazards assumption was tenable. Primary model avoided adjustment for CKM-defining chronic diseases and BMI to prevent overadjustment, while an extended model was used as sensitivity analysis.

#### 2.6.3 Dose–response analysis

Restricted cubic spline models with four knots (5th, 35th, 65th and 95th percentiles) were fitted to examine potential non-linear dose–response relationships, using the median dietary index score as the reference value. Non-linearity was tested by comparing the spline model with the corresponding linear model using likelihood ratio tests (P-nonlinear). All spline models were adjusted for the same covariates as Model 2, ensuring that estimated dose–response relationships account for potential confounding. Linear trends were tested by modeling the median value of each quartile as a continuous variable.

### 2.7 Subgroup and interaction analyses

Subgroup analyses were pre-specified by CKM stage, age, sex, ethnicity, education, smoking, drinking, exercise, and sleep quality. Interaction between CKM stage and dietary indices was evaluated using multiplicative interaction terms in the multivariable model, with statistical significance assessed by likelihood ratio tests.

#### 2.7.1 Sensitivity analyses

All sensitivity analyses were pre-specified in the analysis plan and were conducted to examine the robustness of the main findings. We repeated the analyses after excluding deaths occurring within the first three years of follow-up, participants classified as CKM stage 2 solely because of dyslipidaemia or chronic nephritis, participants with cancer at baseline, and strict vegetarians. In addition, we fitted an extended multivariable Cox model that further adjusted for baseline chronic diseases, including hypertension, diabetes, dyslipidaemia, chronic nephritis and BMI. Because these variables contribute to the CKM stage definition, this extended model was intended solely as a robustness check rather than as the primary analytical model. Stratified analyses were also performed according to age, sex, CKM stage, and exercise status to assess the consistency of the associations across clinically relevant subgroups. In addition, because the comparison of excluded and included participants indicated systematic differences (Supplementary Table 12), we performed inverse probability-of-selection weighting (IPSW): a logistic regression model for inclusion in the analytic sample was fitted on the pooled sample of excluded and included participants using all shared baseline covariates, and stabilized weights (mean 0.98; range 0.39–5.75) were applied in Cox models with robust standard errors (Supplementary Table 16). All sensitivity analyses used the same covariate adjustment as the primary multivariable model unless otherwise specified. The robustness of the findings was judged by the consistency of the direction and magnitude of the hazard ratios across these analyses.

#### 2.7.2 Survival analysis

Kaplan–Meier survival curves stratified by dietary index quartiles and CKM stage were plotted, and differences were compared using the log-rank test.

### 2.8 Statistical software and significance

All analyses were performed using R (version 4.3.1) and Stata 17.0. Two-sided *p*-values <0.05 were considered statistically significant. For subgroup analyses involving multiple comparisons, Bonferroni-adjusted *p*-values were reported; with 18 subgroup strata, the Bonferroni-corrected significance threshold was 0.0028 (0.05/18).

## 3 Result

### 3.1 Participant selection

A total of 16,954 participants from the 2008 wave of the CLHLS were initially considered for inclusion. Participants younger than 65 years (n = 396) were excluded, resulting in 16,558 age-eligible individuals. Subsequently, 9,295 participants with missing baseline diagnoses of hypertension, diabetes, heart disease, coronary heart disease/stroke, dyslipidemia, or chronic nephritis were excluded, leaving 7,263 participants with complete diagnostic data. An additional 166 participants were excluded due to missing follow-up, yielding 7,097 participants with complete follow-up and covariates. Participants with prevalent heart disease or coronary heart disease/stroke at baseline (n = 1,225) were further excluded, resulting in a final analytic sample of 5,872 participants. Within this cohort, participants were classified according to baseline CKM stage: 3,736 in CKM stage 0, 811 in CKM stage 1, and 1,325 in CKM stage 2. During the follow-up period, 2,870 deaths occurred, with a median follow-up duration of 108 months (Figure 1).

A total of 5,872 participants with data-adapted CKM stages 0–2 were included, comprising 3,736 participants with CKM stage 0, 811 with CKM stage 1, and 1,325 with CKM stage 2. Significant differences across CKM stages were observed for age, BMI, sleep duration, alcohol drinking, PDI, hPDI, uPDI, ethnicity, education, and regular exercise. Participants with CKM stage 1 had the highest median BMI and the highest proportion of regular exercise, whereas participants with CKM stage 0 were older, had a higher median uPDI score, and had a slightly lower median PDI score (50.0 versus 51.0 in stages 1 and 2). Sex distribution, household income, and smoking status did not differ significantly across CKM stages (Table 1).

**Table 1:** Patient demographics and baseline characteristics.

Characteristic	CKM			*p*-value
	Stage 0 *N* = 3,736	Stage 1 *N* = 811	Stage 2 *N* = 1,325	
Age, Median (Q1, Q3)	85.0 (75.0, 93.0)	77.0 (70.0, 88.0)	81.0 (73.0, 89.0)	<0.001^1^
Age, Mean ± SD	85 ± 11	80 ± 11	81 ± 10	
Sex, *n* (%)				0.112^2^
Male	1,691 (45.3%)	397 (49.0%)	591 (44.6%)	
Female	2,045 (54.7%)	414 (51.0%)	734 (55.4%)	
BMI, Median (Q1, Q3)	19.5 (17.7, 21.4)	25.4 (24.6, 27.1)	20.2 (18.4, 22.1)	<0.001^1^
Income, Median (Q1, Q3)	10,000 (4,000, 24,000)	10,000 (4,800, 27,000)	10,000 (4,000, 26,000)	0.082^1^
Sleep.hour, Median (Q1, Q3)	8.0 (6.0, 10.0)	8.0 (7.0, 10.0)	8.0 (6.0, 9.0)	0.010^1^
Sleep.quality, *n* (%)				
Bad	340 (9.1%)	65 (8.0%)	118 (8.9%)	
Good	2,001 (53.6%)	455 (56.1%)	671 (50.6%)	
So-so	853 (22.9%)	150 (18.4%)	342 (25.8%)	
Very bad	24 (0.6%)	6 (0.7%)	16 (1.2%)	
Very good	518 (13.9%)	135 (16.6%)	178 (13.4%)	
Drinking, *n* (%)	781 (20.9%)	191 (23.6%)	242 (18.3%)	0.012^2^
Smoking, *n* (%)	795 (21.3%)	168 (20.7%)	261 (19.7%)	0.474^2^
uPDI, Median (Q1, Q3)	51.0 (47.0, 55.0)	48.0 (44.0, 53.0)	50.0 (46.0, 54.0)	<0.001^1^
hpdi, Median (Q1, Q3)	46.0 (42.0, 49.0)	47.0 (43.0, 50.0)	46.0 (43.0, 50.0)	<0.001^1^
PDI, Median (Q1, Q3)	50.0 (45.0, 54.0)	51.0 (48.0, 55.0)	51.0 (47.0, 54.0)	<0.001^1^
Ethnic, *n* (%)				<0.001^2^
Han	3,462 (92.7%)	780 (96.2%)	1,265 (95.5%)	
Others	274 (7.3%)	31 (3.8%)	60 (4.5%)	
Marital, *n* (%)				
Currently married and living with spouse	1,385 (37.1%)	429 (52.9%)	576 (43.5%)	
Divorced	6 (0.2%)	5 (0.6%)	1 (0.1%)	
Never married	33 (0.9%)	5 (0.6%)	9 (0.7%)	
Separated	72 (1.9%)	10 (1.2%)	36 (2.7%)	
Widowed	2,240 (60.0%)	362 (44.6%)	703 (53.1%)	
Education, *n* (%)				<0.001^2^
Educated	1,503 (40.2%)	416 (51.3%)	564 (42.6%)	
No education	2,233 (59.8%)	395 (48.7%)	761 (57.4%)	
Exercise, *n* (%)	1,082 (29.0%)	344 (42.4%)	463 (34.9%)	<0.001^2^
Occupation, *n* (%)				
Agriculture, forestry, animal husbandry or fishery worker	2,776 (74.3%)	482 (59.4%)	902 (68.1%)	
Commercial, service or industrial worker	358 (9.6%)	149 (18.4%)	165 (12.5%)	
Governmental, institutional or managerial personnel	69 (1.8%)	40 (4.9%)	51 (3.8%)	
House worker	222 (5.9%)	49 (6.0%)	84 (6.3%)	
Military personnel	28 (0.7%)	8 (1.0%)	7 (0.5%)	
Never worked	18 (0.5%)	6 (0.7%)	6 (0.5%)	
Others	68 (1.8%)	10 (1.2%)	32 (2.4%)	
Professional and technical personnel	125 (3.3%)	52 (6.4%)	53 (4.0%)	
Self-employed	72 (1.9%)	15 (1.8%)	25 (1.9%)	

1 Kruskal-Wallis rank sum test.

2 Pearson’s Chi-squared test.

The associations of PDI, hPDI and uPDI with all-cause mortality are presented in Table 2. In the unadjusted model, higher hPDI was associated with a lower risk of all-cause mortality, whereas higher uPDI was associated with a higher risk. After multivariable adjustment, hPDI remained inversely associated with all-cause mortality when analyzed as a continuous variable (HR = 0.989, 95% CI: 0.982–0.995; *p* < 0.001). Compared with participants in the lowest hPDI quartile, those in the highest quartile had a significantly lower risk of all-cause mortality (HR = 0.868, 95% CI: 0.783–0.961; *p* = 0.007), with a significant trend across quartiles (P for trend = 0.020). Conversely, uPDI was positively associated with all-cause mortality in the fully adjusted model (HR = 1.012, 95% CI: 1.006–1.019; *p* < 0.001). Participants in the highest uPDI quartile had a higher risk of all-cause mortality than those in the lowest quartile (HR = 1.192, 95% CI: 1.060–1.340; *p* = 0.003), with a significant dose–response trend across quartiles (P for trend = 0.006). Regarding the overall PDI, the unadjusted model showed a lower risk of all-cause mortality per 1-point increase (HR = 0.982, 95% CI: 0.976–0.988; *p* < 0.001), with the highest quartile having a 25.1% lower risk than the lowest quartile (HR = 0.749, 95% CI: 0.677–0.830; P for trend < 0.001). However, this association was abolished after multivariable adjustment (continuous HR = 1.001, 95% CI: 0.994–1.007; Q4 vs. Q1: HR = 1.067, 95% CI: 0.956–1.191; P for trend = 0.584) (Table 2; Supplementary Table 13). Restricted cubic spline analysis confirmed the absence of an independent dose–response association for PDI (P-overall = 0.887; P-nonlinear = 0.749; reference = median of 50) (Supplementary Figure 1). The null adjusted association was consistent across all sensitivity analyses (Supplementary Table 14) and across all subgroup strata (all Bonferroni-corrected *p* > 0.05; Supplementary Table 15).

**Table 2:** Association between PDI, hPDI/uPDI and all-cause mortality (Cox regression).

Characteristic	*N*	Event *N*	Model 1			Model 2		
			HR	95% CI	*p*-value	HR	95% CI	*p*-value
hPDI (continuous)	5,872	2,870	0.982	0.976, 0.989	<0.001	0.989	0.982, 0.995	<0.001
hPDI								
Q1 [26, 42]	1,289	707	—	—		—	—	
Q2 [42, 46]	1,438	690	0.867	0.781, 0.963	0.008	0.917	0.825, 1.020	0.111
Q3 [46, 49]	1,309	642	0.896	0.805, 0.998	0.045	0.972	0.872, 1.083	0.607
Q4 [49, 71]	1,836	831	0.789	0.713, 0.872	<0.001	0.868	0.783, 0.961	0.007
*p* for trend					<0.001			0.020
uPDI (continuous)	5,872	2,870	1.020	1.014, 1.026	<0.001	1.012	1.006, 1.019	<0.001
uPDI								
Q1 [25, 46]	1,304	515	—	—		—	—	
Q2 [46, 50]	1,306	638	1.217	1.083, 1.367	<0.001	1.103	0.979, 1.242	0.107
Q3 [50, 55]	1,733	855	1.216	1.090, 1.356	<0.001	1.097	0.978, 1.230	0.115
Q4 [55, 71]	1,529	862	1.438	1.289, 1.604	<0.001	1.192	1.060, 1.340	0.003
*p* for trend					<0.001			0.006
PDI (continuous)	5,872	2,870	0.982	0.976, 0.988	<0.001	1.001	0.994, 1.007	0.798
PDI								
Q1 [27, 46]	1,293	721	—	—		—	—	
Q2 [46, 50]	1,279	626	0.908	0.816, 1.011	0.077	0.970	0.870, 1.082	0.582
Q3 [50, 54]	1,540	758	0.897	0.810, 0.994	0.037	1.006	0.905, 1.120	0.907
Q4 [54, 70]	1,759	764	0.749	0.677, 0.830	<0.001	1.067	0.956, 1.191	0.245
*p* for trend					<0.001			0.584

CI, Confidence Interval; HR, Hazard Ratio.

Model 1: no covariates were adjusted.

Model 2: adjusted for age, sex, ethnic, marital, education, smoking, drinking, income, exercise, occupation, sleep.hour, and sleep.quality.

Restricted cubic spline analyses further supported these associations (Figure 2). The association between hPDI and all-cause mortality was statistically significant overall (P-overall = 0.008), with no evidence of a nonlinear relationship (P-nonlinear = 0.224), suggesting an approximately linear inverse association. In contrast, uPDI showed a significant positive association with all-cause mortality (P-overall = 0.011), also without evidence of nonlinearity (P-nonlinear = 0.557). The confidence intervals were wider at the extreme ends of the dietary index distributions, likely reflecting the smaller number of participants with very low or very high scores.

**Figure 2:**
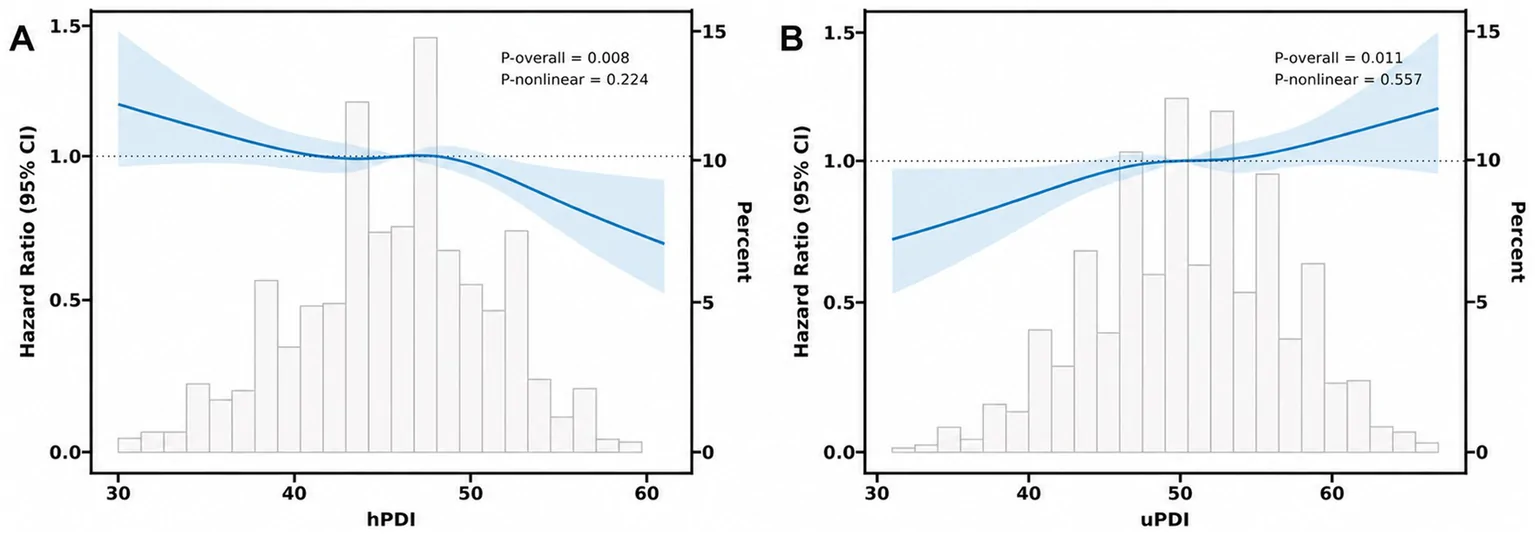
Restricted cubic spline analyses of the associations of hpDI and uPDI with all-cause mortality. Panel **A** shows the dose-response association between hpDI and all-cause mortality, and Panel **B** shows the corresponding association for uPDI. Solid blue lines represent adjusted hazard ratios, and shaded areas indicate 95% confidence intervals. Histograms show the distribution of dietary index scores. The reference value was the median dieatary index score. Models were adjusted for age, sex, ethnicity, martial status, education, smoking, alcohol consumption, household income, physical activity, occupation, sleep duration, and sleep quality, hDPI, healthful plant-based diet index; uPDI, unhealthful plant-based diet index; CI, confidence interval.

In the unadjusted model (in Table 3), hPDI was associated with a lower risk of mortality across all CKM groups, whereas uPDI was associated with a higher risk of mortality. After multivariable adjustment, the inverse association between hPDI and mortality remained significant in CKM stages 1 and 2, but not in CKM stage 0. Similarly, uPDI remained positively associated with mortality in CKM stages 1 and 2, whereas the association was attenuated and became non-significant in CKM stage 0. However, within-stratum significance does not imply a significant between-stratum interaction: the formal interaction test between CKM status and hPDI was only marginally significant in the unadjusted model (*p* = 0.012) and not significant after multivariable adjustment (*p* = 0.062), and no significant interaction was observed for uPDI (adjusted *p* = 0.824). Accordingly, although the stratified estimates suggest numerically stronger associations in CKM stages 1–2, formal evidence for effect modification by CKM stage remains limited. For the overall PDI, the unadjusted estimates were inversely associated with mortality across all CKM stages, but the associations were null after multivariable adjustment in all three stages (Table 3); no significant PDI-by-CKM interaction was observed (adjusted *p* = 0.242).

**Table 3:** Association between PDI, hPDI/uPDI and all-cause mortality in CKM 0–2 stages (Cox risk regression).

Characteristic	*N*	Model 1			Model 2		
		HR 95% CI	*p*-value	*p* for interaction	HR 95% CI	*p*-value	*p* for interaction
CKM (hPDI)				0.012			0.062
Stage 0	3,736	0.989 (0.981–0.997)	0.006		0.994 (0.986–1.002)	0.116	
Stage 1	811	0.955 (0.934–0.977)	<0.001		0.961 (0.938–0.985)	0.001	
Stage 2	1,325	0.982 (0.968–0.996)	0.013		0.983 (0.968–0.997)	0.022	
CKM (uPDI)				0.104			0.824
Stage 0	3,736	1.013 (1.006–1.021)	0.001		1.005 (0.997–1.014)	0.179	
Stage 1	811	1.034 (1.015–1.054)	<0.001		1.023 (1.002–1.044)	0.034	
Stage 2	1,325	1.021 (1.009–1.034)	0.001		1.015 (1.000–1.029)	0.049	
CKM (PDI)				0.926			0.242
Stage 0	3,736	0.986 (0.979–0.992)	<0.001		1.000 (0.992–1.007)	0.921	
Stage 1	811	0.958 (0.937–0.979)	<0.001		0.990 (0.968–1.013)	0.403	
Stage 2	1,325	0.991 (0.977–1.005)	0.190		1.009 (0.994–1.024)	0.226	

CI, Confidence Interval; HR, Hazard Ratio.

Model 1: no covariates were adjusted.

Model 2: adjusted for age, sex, ethnic, marital, education, smoking, drinking, income, exercise, occupation, sleep.hour, and sleep.quality.

The Kaplan–Meier survival curve showed that within different CKM stages, the survival probabilities of the hPDI and uPDI quartile groups vary to varying degrees (Figure 3). For hPDI, there was a statistically significant difference in the survival curves of different quartile groups among the CKM stage 1 and stage 2 populations, but not among the CKM stage 0 population. Overall, the survival probabilities of the higher hPDI quartile groups were relatively higher, with numerically larger separations in CKM stages 1 and 2. For PDI, crude differences across quartiles were also observed (log-rank *p* < 0.001), consistent with the unadjusted Cox estimates (Supplementary Figure 2). For uPDI, higher quartiles were generally associated with lower survival probabilities, particularly among participants with CKM stages 1 and 2.

**Figure 3:**
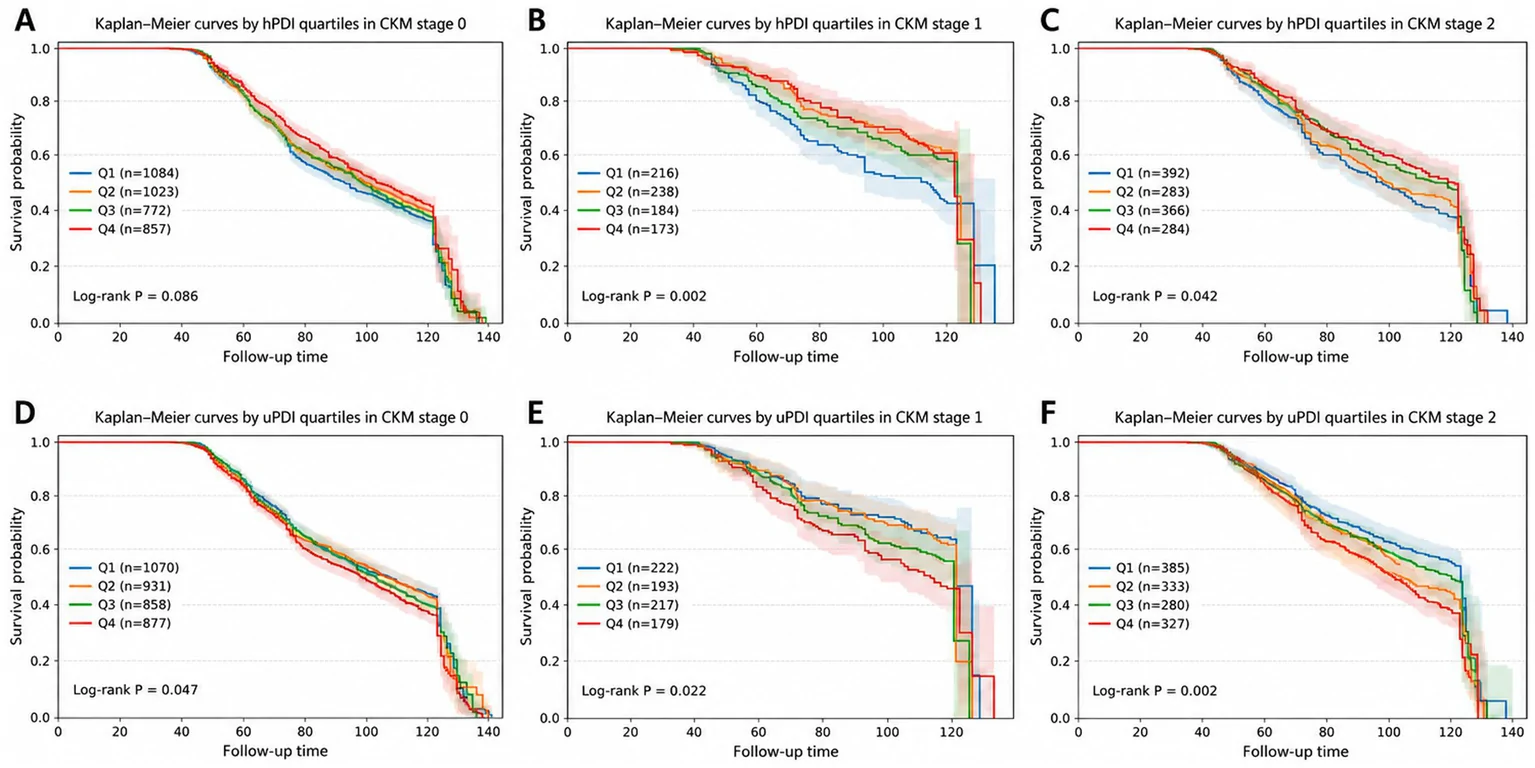
Kaplan–Meier survival curves for all-cause mortality according to quartiles of hPDI and uPDI across CKM stages 0-2. Panels **A–C** show Kaplan–Meier curves by hPDI quartiles in CKM stage 0, CKM stage 1, and CKM stage 2, respectively. Panels **D–F** show Kaplan–Meier curves by uPDI quartiles in CKM stage 0, CKM stage 1, and CKM stage 2, respectively. Survival differences across quartiles were compared using the log-rank test. hPDI, healthy plant-based diet index; uPDI, unhealthy plant-based diet index; CKM, cardiovascular kidney-metabolic.

### 3.2 Subgroup analysis

Subgroup analyses showed broadly consistent associations of hPDI and uPDI with all-cause mortality across predefined strata (Supplementary Tables 4, 5). For hPDI, the inverse association was observed in the overall population (HR = 0.989, 95% CI: 0.982–0.995) and remained significant after Bonferroni correction in several subgroups, including men, women, Han participants, participants with or without education, non-smokers, non-drinkers, those with regular exercise, and those with good sleep quality. A significant interaction was observed only for regular exercise (P for interaction = 0.001), with a stronger inverse association among participants who exercised regularly. For uPDI, the positive association with mortality was observed overall (HR = 1.012, 95% CI: 1.006–1.019) and remained significant after correction across most sex and lifestyle subgroups. No significant interaction was observed for uPDI. These exploratory subgroup findings should be interpreted cautiously, with emphasis placed on formal interaction tests rather than within-stratum significance. For the overall PDI, no subgroup stratum remained significant after Bonferroni correction (all corrected *p* > 0.05); nominally significant interactions were observed only for sex (*p* = 0.008), education (*p* = 0.014), and exercise (*p* = 0.004) (Supplementary Table 15).

### 3.3 Sensitivity analyses

The associations were largely unchanged across sensitivity analyses. After excluding deaths within the first 3 years of follow-up, excluding participants classified as CKM stage 2 solely because of dyslipidaemia or chronic nephritis, excluding participants with cancer at baseline, and excluding strict vegetarians, higher hPDI remained associated with lower all-cause mortality, whereas higher uPDI remained associated with higher mortality. In the extended model additionally adjusted for hypertension, diabetes, dyslipidaemia, and chronic nephritis, the associations were also preserved, with the highest hPDI quartile associated with lower mortality risk (HR = 0.867, 95% CI: 0.782–0.961) and the highest uPDI quartile associated with higher mortality risk (HR = 1.186, 95% CI: 1.054–1.334). Stratified analyses by age, sex, CKM stage, and exercise status showed generally consistent directions of association, although several subgroup estimates were less precise. These results indicate that the main findings were robust to alternative analytic assumptions and sample restrictions (Supplementary Tables 6–11). The null adjusted associations of the overall PDI were likewise stable across all sensitivity analyses (Supplementary Table 14). In the inverse probability-of-selection-weighted analyses, the directions of association in the unadjusted models were consistent with those observed in the primary analyses. Higher hPDI was associated with lower all-cause mortality (HR = 0.980, 95% CI: 0.974–0.986), whereas higher uPDI was associated with higher mortality (HR = 1.018, 95% CI: 1.012–1.024); PDI was also inversely associated with mortality (HR = 0.981, 95% CI: 0.976–0.987) (Supplementary Table 16). After further adjustment for relevant covariates, the directions of association remained consistent, although the effect estimates were attenuated toward the null and the corresponding confidence intervals included unity (Supplementary Table 16).

## 4 Discussion

In this prospective cohort study of older Chinese adults classified as having data-adapted CKM stages 0–2, we observed divergent associations between the quality of plant-based dietary patterns and all-cause mortality. Higher adherence to a healthful plant-based diet, as reflected by hPDI, was associated with lower mortality risk, whereas higher adherence to an unhealthful plant-based diet, as reflected by uPDI, was associated with higher mortality risk. Importantly, the findings were generally robust across multiple sensitivity analyses, including exclusion of early deaths, exclusion of participants with baseline cancer, exclusion of strict vegetarians, alternative CKM stage 2 definitions, and additional adjustment for baseline chronic diseases.

The numerically stronger stratified estimates in CKM stages 1–2 should be interpreted cautiously given the absence of statistically significant interaction; if confirmed in future studies, such a pattern may be biologically plausible. CKM stage 1 is characterized by excess adiposity or abdominal obesity, while CKM stage 2 reflects the presence of metabolic or kidney-related risk factors such as hypertension, diabetes, dyslipidaemia, or chronic nephritis. These early stages represent a period in which metabolic dysregulation, inflammation, endothelial dysfunction, insulin resistance, and renal stress may already be present but may still be modifiable (19–21). Healthful plant-based diets are typically rich in fiber, antioxidants, unsaturated fatty acids, minerals, and phytochemicals, which may improve glycaemic control, lipid metabolism, blood pressure, systemic inflammation, oxidative stress, and gut microbiota composition (22, 23). These pathways are closely related to CKM progression and mortality risk. Conversely, unhealthful plant-based diets characterized by refined carbohydrates, sugar, and preserved foods may worsen glycaemic fluctuation, sodium load, inflammation, and metabolic dysfunction, thereby contributing to higher mortality risk among individuals with early CKM abnormalities (24, 25).

At the molecular level, accumulating evidence indicates that the pathophysiology of CKM syndrome involves activation of oxidative stress and chronic inflammation, dysregulation of the renin–angiotensin system, aberrant Wnt1/β-catenin signaling, and gut microbiota imbalance (26–30). Recent reviews of the therapeutic approach to CKM syndrome and of updated KDIGO guidelines further emphasize the systemic interactions among these pathways (26, 27). Gut microbial-derived metabolites have emerged as key mediators of kidney injury and fibrosis, and targeting the gut microbiota is increasingly regarded as a promising strategy for chronic kidney disease (31–33). Healthful plant-based diets, which are rich in dietary fiber, polyphenols, and unsaturated fatty acids, may counter these pathways by modulating the gut microbiota, reducing oxidative stress and inflammation, and improving endothelial and metabolic function, thereby providing a plausible biological basis for the observed survival benefit (33).

In contrast, the associations between plant-based diet indices and mortality were less apparent among participants with CKM stage 0. Several explanations are possible. First, individuals in data-adapted CKM stage 0 had relatively favorable cardiometabolic profiles based on the available anthropometric and self-reported clinical information, leaving less room for dietary modification to produce measurable short- to medium-term mortality benefits. Second, among very old adults, mortality may be influenced by frailty, infection, functional decline, or other non-cardiometabolic causes, which could attenuate the apparent contribution of diet-related cardiometabolic pathways. Third, because data-adapted CKM stage 0 participants had no self-reported or anthropometrically detected major cardiometabolic risk factors at baseline, the absolute risk reduction associated with dietary improvement may be smaller than that in higher-risk individuals. Therefore, the null or weaker associations in CKM stage 0 should not be interpreted as evidence that diet is unimportant in this group, but rather as suggesting that the mortality benefit of plant-based dietary quality may be more detectable among individuals with established early cardiometabolic risk.

The sensitivity analyses further support the robustness of the main findings. After excluding deaths within the first 3 years of follow-up, the highest hPDI quartile remained associated with lower mortality risk, whereas the highest uPDI quartile remained associated with higher mortality risk, reducing concern that the observed associations were primarily driven by reverse causation. Similar patterns were observed after excluding participants with baseline cancer and after excluding strict vegetarians, suggesting that the findings were not solely attributable to pre-existing severe disease or extreme dietary behavior. Moreover, when participants classified as CKM stage 2 solely because of dyslipidaemia or chronic nephritis were excluded, the direction of association remained largely unchanged, indicating that the results were not dependent on a single operational definition of CKM stage 2. Additional adjustment for hypertension, diabetes, dyslipidaemia, and chronic nephritis also did not materially alter the associations, although the effect estimates were modestly attenuated. This is important because these chronic conditions may act both as confounders and as intermediate factors linking diet to mortality. The persistence of the associations after this additional adjustment suggests that plant-based dietary quality may capture broader health-related dietary exposures beyond the presence of baseline chronic disease alone.

The stratified analyses also provide useful context. The inverse association between hPDI and mortality and the positive association between uPDI and mortality were generally consistent across age, sex, CKM stage, and exercise subgroups, although some estimates were attenuated or imprecise. Such imprecision is expected because subgroup analyses reduce sample size and statistical power. Notably, the association of hPDI appeared more evident among participants with regular exercise, suggesting that healthy diet and physical activity may have complementary roles in reducing mortality risk. However, these subgroup findings should be interpreted cautiously, especially when formal interaction evidence is limited. Rather than indicating that dietary interventions are only useful in selected subgroups, the overall pattern suggests that plant-based dietary quality may be broadly relevant, with potentially greater benefit among individuals who already engage in healthier lifestyle behaviors or who have early cardiometabolic risk.

From a public health perspective, our findings emphasize the importance of dietary quality in the prevention and management of early CKM syndrome. For older adults with data-adapted early-stage CKM syndrome, promoting a healthful plant-based dietary pattern may represent a feasible component of risk management. Although the stratified estimates appeared numerically stronger in CKM stages 1–2, formal interaction tests did not establish differential associations across CKM stages. This does not necessarily imply strict vegetarianism; rather, it supports a dietary pattern characterized by higher consumption of whole grains, fresh vegetables, fruits, legumes, nuts, and tea, together with lower consumption of refined grains, sugar-rich foods, preserved vegetables, and excessive animal-based foods. This distinction is especially relevant in China, where traditional diets may include both healthful plant foods and high-sodium preserved plant foods. Therefore, dietary guidance for older adults with early CKM should focus not only on increasing plant food intake, but also on improving the quality and composition of plant-based foods.

Notably, the overall PDI was not associated with mortality after multivariable adjustment in this study, in contrast to the significant associations observed for hPDI and uPDI. This contrast supports the concept that the mere quantity of plant-based foods, irrespective of their quality, may not be sufficient to confer survival benefits, and is consistent with the present findings that healthful and unhealthful plant foods have divergent associations with mortality. These results therefore reinforce the message that dietary recommendations for early CKM management should emphasize the quality of plant-based foods. The three indices were moderately or weakly intercorrelated (Pearson r: PDI–hPDI 0.63, PDI–uPDI −0.11, hPDI–uPDI −0.18; Spearman ρ: 0.59, −0.11, −0.15; Supplementary Table 17), indicating that they capture related but distinct dimensions of plant-based eating, which further supports the interpretation that dietary quality, rather than the overall degree of plant-based eating, is the principal signal observed in this study.

This study has several strengths. It used data from a large, community-based cohort of older Chinese adults with long-term mortality follow-up, enabling evaluation of diet–mortality associations in a population at high absolute risk. The study also applied the CKM staging framework to distinguish individuals at different stages of cardiometabolic and kidney-related risk, providing a more clinically meaningful context than analyses based solely on single diseases. In addition, both hPDI and uPDI were examined, allowing us to differentiate healthful from unhealthful plant-based dietary patterns. Multiple sensitivity analyses were performed to assess the influence of reverse causation, baseline cancer, strict vegetarian dietary patterns, CKM stage 2 classification, and baseline chronic diseases, and the results were generally consistent.

Several limitations should also be acknowledged. First, the CKM staging was based on a data-adapted operational definition rather than the full biomarker-based CKM criteria. Because the CLHLS lacked laboratory measures of kidney function, albuminuria, lipid profiles, and glycaemic biomarkers, CKM stages were classified using anthropometric indicators and self-reported physician-diagnosed conditions. This may have introduced some misclassification, particularly for undiagnosed kidney dysfunction, dyslipidaemia, or glucose abnormalities. However, the main findings remained consistent after excluding participants whose CKM stage 2 classification was based solely on dyslipidaemia or chronic nephritis, and after additional adjustment for baseline chronic diseases. These results suggest that the observed associations between plant-based diet quality and mortality were unlikely to be driven solely by the adapted CKM definition; nevertheless, they do not establish equivalence with the full biomarker-based AHA classification, and whether these findings extend to populations classified using complete biomarker-based CKM criteria requires confirmation in future studies. Moreover, misclassification arising from self-reported diagnoses is expected to be largely non-differential with respect to mortality, which would generally attenuate associations toward the null; participants with undiagnosed metabolic or kidney abnormalities may thus have been assigned to CKM stage 0, potentially contributing to the weaker associations observed in that stage. Second, dietary intake was assessed using self-reported frequency data rather than detailed quantitative dietary assessment, which may introduce measurement error; because the food frequency questionnaire recorded consumption frequency without portion sizes, a sensitivity analysis adjusting for total energy intake could not be performed. Although physical activity was included as a covariate, this does not resolve this limitation, and residual confounding by total energy balance cannot be excluded. In addition, diet was assessed only once at baseline, and dietary habits may have changed during the approximately nine-year follow-up, potentially leading to exposure misclassification and attenuation of the observed associations. Third, residual confounding cannot be excluded, despite adjustment for major demographic, lifestyle, and clinical covariates. In particular, information on medication use, frailty, functional status (e.g., activities of daily living), cognitive function, and total energy intake was not available in the current data extract, and these unmeasured factors may represent additional sources of residual confounding. Fourth, cause-of-death information was not available in the current data extract; therefore, cause-specific mortality, especially cardiovascular and kidney-related mortality, could not be evaluated. Given the mechanistic relevance of cardiorenal pathways to both diet and CKM syndrome, cause-specific analyses in cohorts with complete death-registry linkage represent an important direction for future research. Fifth, the study population consisted of older Chinese adults, and the findings may not be directly generalisable to younger populations or to populations with different dietary structures and healthcare contexts. In addition, although sensitivity analyses supported the robustness of the findings, the observational design precludes causal inference, and reverse causation—whereby preclinical disease influenced dietary habits at baseline—cannot be entirely excluded despite the exclusion of deaths occurring within the first 3 years. Finally, selection bias cannot be fully ruled out: participants excluded because of missing diagnostic data (n = 9,295) were older (median age 92 vs. 83 years) and had less healthful diets (median hPDI 44 vs. 46; median uPDI 53 vs. 50) than the analytical sample (Supplementary Table 12). This differential exclusion may have affected both the distribution of dietary exposures and the magnitude of the observed associations in the analytic sample, rather than only limiting generalizability; the direction of selection-related bias is difficult to predict, although the exclusion of older participants with less healthful diets may plausibly have attenuated the observed associations. Consistent with this concern, the inverse probability-of-selection-weighted estimates remained directionally similar to the primary analyses but were attenuated toward the null, suggesting that selection related to missing diagnostic information may have influenced the magnitude of the observed associations.

## 5 Conclusion

Among older Chinese adults with data-adapted early-stage CKM syndrome, higher hPDI was associated with lower all-cause mortality, whereas higher uPDI was associated with higher mortality. These findings highlight that the quality of plant-based foods, rather than plant-based eating per se, may be important for mortality risk management in early CKM syndrome. The stratified estimates suggested numerically stronger associations in CKM stages 1–2, but formal interaction evidence remained limited. Future studies using comprehensive biomarker-based CKM definitions are needed to validate these results.

## Data Availability

The original contributions presented in the study are included in the article/Supplementary material, further inquiries can be directed to the corresponding authors.
