# Supplementary material for: The associations between healthful and unhealthful plant-based dietary patterns and all-cause mortality among older Chinese adults with data-adapted early-stage cardiovascular-kidney-metabolic syndrome (stages 0–2): a prospective cohort study from the CLHLS
Source: Front Nutr. 2026 Oct 16;13:1915953. doi: 10.3389/fnut.2026.1915953 (PMC13721378; doi:10.3389/fnut.2026.1915953)
Supplement: Supplementary file 1 [file Supplementary_file_1.docx]

Catalogue

[Supplementary Table 1. Definition of baseline information and relevant covariates	2](#_Toc10488)

[Supplementary Table 2. Percentages of missingness	3](#_Toc6983)

[Supplementary Table 3. Food group classification and scoring criteria for PDI, hPDI, and uPDI	4](#_Toc10966)

[Supplementary Table 4. Subgroup analysis between hPDI and all-cause mortality in CKM 0-2.	6](#_Toc29222)

[Supplementary Table 5. Subgroup analysis between uPDI and all-cause mortality in CKM 0-2.	8](#_Toc32239)

[Supplementary Table 6. Associations of plant-based diets with all-cause mortality by excluding deaths within the first three years of follow-up.	10](#_Toc10842)

[Supplementary Table 7. To explore the association between a plant-based diet and mortality in CKM 0-2 stage (excluding participants who were classified as stage 2 due to dyslipidemia or chronic nephritis)	11](#_Toc5878)

[Supplementary Table 8. Exclusion of baseline cancer participants to investigate the association between plant-based diet and all-cause mortality in CKM 0-2 stage.	12](#_Toc6140)

[Supplementary Table 9. Exploring the association between plant-based diets and all-cause mortality in CKM 0=2 stages after excluding strict vegetarians.	13](#_Toc11017)

[Supplementary Table 10. Construct a new Cox risk model (model 3) by incorporating chronic diseases as confounding factors and conduct sensitivity analysis.	14](#_Toc12149)

[Supplementary Table 11. Sensitivity analyses stratified by age, sex, CKM stage, and regular exercise.	15](#_Toc10666)

[Supplementary Table 12. Baseline characteristics of participants excluded for missing diagnostic data versus the analytical sample.	18](#_Toc9231)

[Supplementary Table 13. Association between the overall plant-based diet index (PDI) and all-cause mortality in older adults with CKM stages 0-2 .	20](#_Toc15234)

[Supplementary Table 13b. Association between PDI (continuous, per 1-point increase) and all-cause mortality by CKM stage.	21](#_Toc23603)

[Supplementary Table 14. Sensitivity analyses for the association between PDI and all-cause mortality in CKM stages 0-2 (new in this revision).	22](#_Toc5172)

[Supplementary Table 15. Subgroup analysis between PDI (continuous, per 1-point increase) and all-cause mortality in CKM stages 0-2 (new in this revision).	25](#_Toc28779)

[Supplementary Table 16. Inverse probability-of-selection-weighted (IPSW) analyses of the associations between dietary indices and all-cause mortality (new in this revision).	26](#_Toc6086)

[Supplementary Table 17. Correlations among PDI, hPDI, and uPDI (new in this revision).	27](#_Toc21410)

[Supplementary Figure 1. Restricted cubic spline for the association between PDI and all-cause mortality (new in this revision).	28](#_Toc6260)

[Supplementary Figure 2. Kaplan-Meier survival curves for all-cause mortality according to PDI quartiles, overall and by CKM stage (new in this revision).	29](#_Toc30267)

# **Supplementary Table 1. Definition of baseline information and relevant covariates**

| **Covariate** | **Definition and coding** | |
| --- | --- | --- |
| Age | Age at baseline, analysed as a continuous variable. | v_bthyr |
| Sex | Categorised as male or female. | a1 |
| Ethnic | Categorised as Han Chinese or other ethnic groups. | a2 |
| Marital status | Categorised as currently married and living with spouse, widowed, divorced, separated, or never married. | f41 |
| Education | Dichotomised as having received formal education or no formal education. | f1_11 |
| Household income | Self-reported annual household income, recorded in Chinese yuan and analysed as a continuous variable. | f35_11 |
| Occupation | Categorised according to the participant’s main lifetime occupation, including agriculture, forestry, animal husbandry or fishery worker; commercial, service or industrial worker; governmental, institutional or managerial personnel; professional and technical personnel; house worker; military personnel; self-employed; never worked; and other occupations. | f2_11 |
| Smoking status | Defined as current smoking versus non-current smoking. | d71_11 |
| Drinking consumption | Defined as current drinking versus non-drinking. | d81_11 |
| Regular physical activity | Defined according to whether participants reported exercising regularly at present; categorised as yes or no. | d91_11 |
| Body mass index | Calculated as weight in kilograms divided by height in metres squared and analysed as a continuous variable. | g1021_11,g101_11 |
| Sleep duration | Self-reported average number of hours of sleep per day, analysed as a continuous variable. | g02_11 |
| Sleep quality | Self-reported sleep quality, categorised as very good, good, fair/so-so, bad, or very bad. | g01_11 |
| Hypertension | Self-reported doctor-diagnosed hypertension; additionally adjusted for in sensitivity analyses. | g15a11_11 |
| Diabetes | Self-reported doctor-diagnosed diabetes; additionally adjusted for in sensitivity analyses. | g15b1_11 |
| Dyslipidaemia | Self-reported doctor-diagnosed dyslipidaemia; additionally adjusted for in sensitivity analyses. | g15c1_11 |
| Chronic nephritis | Self-reported doctor-diagnosed chronic nephritis; additionally adjusted for in sensitivity analyses. | g15d1_11 |
| Heart disease | Self-reported doctor-diagnosed heart disease; As the diagnostic criterion for CKM stage 4 | g15s1_11 |
| CVD/Stroke | Self-reported doctor-diagnosed CVD/Stroke; As the diagnostic criterion for CKM stage 4 | g15r1_11 |
| Waistline | Calculated as waistline in Centimeter.As the diagnostic criterion for CKM stage 1 | g102c_11 |

# **Supplementary Table 2. Percentages of missingness**

| **Cohorts** | **Covariates** | **Percentage of missingness** |
| --- | --- | --- |
| **CLHLS** | Education | 0.17% |
|  | Ethnic | 0.00% |
|  | Marital status | 0.00% |
|  | Income | 7.10% |
|  | Occupation | 0.01% |
|  | Smoking status | 0.00% |
|  | Drinking status | 0.00% |
|  | Regular exercise | 0.10% |
|  | Sleep.hour | 0.10% |
|  | sleep.quality | 0.18% |
| CLHLS, Chinese Longitudinal Healthy Longevity Survey | | |

# **Supplementary Table 3. Food group classification and scoring criteria for PDI, hPDI, and uPDI**

16 food groups were classified into three categories: (i) healthful plant foods (e.g., whole grains, fresh fruits, fresh vegetables, legumes, nuts, tea); (ii) unhealthful plant foods (e.g., refined grains, preserved vegetables, sugar); and (iii) animal foods (e.g., meat, fish, eggs, dairy). Each food group was scored according to frequency of consumption, ranging from 1 (least frequent) to 5 (most frequent). For the hPDI, positive scores were assigned to healthful plant foods, whereas unhealthful plant foods and animal foods were reverse-scored. For the uPDI, positive scores were assigned to unhealthful plant foods, while healthful plant foods and animal foods were reverse-scored. The total score for each index ranged from 16 to 80, with higher hPDI and lower uPDI indicating a healthier plant-based dietary pattern. For the PDI, all plant food groups received positive scores and all animal food groups received reverse scores, irrespective of food healthfulness.

| **Food category** | **Food** | **Frequency** | **hPDI** | **uPDI** | **PDI** |
| --- | --- | --- | --- | --- | --- |
| Plant food | Whole grain | Yes | 5 | 1 | 5 |
|  |  | No | 1 | 5 | 1 |
|  | Vegetable oil | Yes | 5 | 1 | 5 |
|  |  | No | 1 | 5 | 1 |
|  | Fresh fruit | Almost everyday | 5 | 1 | 5 |
|  |  | Quite often | 4 | 2 | 4 |
|  |  | Occasionally | 2 | 4 | 2 |
|  |  | Rarely or never | 1 | 5 | 1 |
|  | Fresh vegetable | Almost everyday | 5 | 1 | 5 |
|  |  | Quite often | 4 | 2 | 4 |
|  |  | Occasionally | 2 | 4 | 2 |
|  |  | Rarely or never | 1 | 5 | 1 |
|  | Legu-me | Almost everyday | 5 | 1 | 5 |
|  |  | ≥1 time/week | 4 | 2 | 4 |
|  |  | ≥1 time/month | 3 | 3 | 3 |
|  |  | Occasionally | 2 | 4 | 2 |
|  |  | Rarely or never | 1 | 5 | 1 |
|  | Garlic | Almost everyday | 5 | 1 | 5 |
|  |  | ≥1 time/week | 4 | 2 | 4 |
|  |  | ≥1 time/month | 3 | 3 | 3 |
|  |  | Occasionally | 2 | 4 | 2 |
|  |  | Rarely or never | 1 | 5 | 1 |
|  | Nut | Almost everyday | 5 | 1 | 5 |
|  |  | ≥1 time/week | 4 | 2 | 4 |
|  |  | ≥1 time/month | 3 | 3 | 3 |
|  |  | Occasionally | 2 | 4 | 2 |
|  |  | Rarely or never | 1 | 5 | 1 |
|  | Tea | Almost everyday | 5 | 1 | 5 |
|  |  | ≥1 time/week | 4 | 2 | 4 |
|  |  | ≥1 time/month | 3 | 3 | 3 |
|  |  | Occasionally | 2 | 4 | 2 |
|  |  | Rarely or never | 1 | 5 | 1 |
|  | Refined grain | Yes | 1 | 5 | 5 |
|  |  | No | 5 | 1 | 1 |
|  | Sugar | Almost everyday | 1 | 5 | 5 |
|  |  | ≥1 time/week | 2 | 4 | 4 |
|  |  | ≥1 time/month | 3 | 3 | 3 |
|  |  | Occasionally | 4 | 2 | 2 |
|  |  | Rarely or never | 5 | 1 | 1 |
|  | Salt-preserved vegetable | Almost everyday | 1 | 5 | 5 |
|  |  | ≥1 time/week | 2 | 4 | 4 |
|  |  | ≥1 time/month | 3 | 3 | 3 |
|  |  | Occasionally | 4 | 2 | 2 |
|  |  | Rarely or never | 5 | 1 | 1 |
| Animal food | Animal fat | Yes | 1 | 1 | 1 |
|  |  | No | 5 | 5 | 5 |
|  | Meat | Almost everyday | 1 | 1 | 1 |
|  |  | ≥1 time/week | 2 | 2 | 2 |
|  |  | ≥1 time/month | 3 | 3 | 3 |
|  |  | Occasionally | 4 | 4 | 4 |
|  |  | Rarely or never | 5 | 5 | 5 |
|  | Fish | Almost everyday | 1 | 1 | 1 |
|  |  | ≥1 time/week | 2 | 2 | 2 |
|  |  | ≥1 time/month | 3 | 3 | 3 |
|  |  | Occasionally | 4 | 4 | 4 |
|  |  | Rarely or never | 5 | 5 | 5 |
|  | Egg | Almost everyday | 1 | 1 | 1 |
|  |  | ≥1 time/week | 2 | 2 | 2 |
|  |  | ≥1 time/month | 3 | 3 | 3 |
|  |  | Occasionally | 4 | 4 | 4 |
|  |  | Rarely or never | 5 | 5 | 5 |
|  | Dairy products | Almost everyday | 1 | 1 | 1 |
|  |  | ≥1 time/week | 2 | 2 | 2 |
|  |  | ≥1 time/month | 3 | 3 | 3 |
|  |  | Occasionally | 4 | 4 | 4 |
|  |  | Rarely or never | 5 | 5 | 5 |

# **Supplementary Table 4. Subgroup analysis between hPDI and all-cause mortality in CKM 0-2.**

| **Subgroup** | **N** | **Adjusted HR (95% CI)** | **P value** | **Bonferroni-adjusted P value** | **P for interaction** |
| --- | --- | --- | --- | --- | --- |
| **Overall** | 5872 | 0.989 (0.982-0.995) | <0.001 |  |  |
| **Age** |  |  |  |  | 0.389 |
| ˂75 | 1756 | 0.996 (0.977-1.016) | 0.724 | 1.000 |  |
| ˃84 | 2701 | 1.001 (0.993-1.009) | 0.82 | 1.000 |  |
| 75 to 84 | 1415 | 0.996 (0.981-1.010) | 0.546 | 1.000 |  |
| **Sex** |  |  |  |  | 0.86 |
| male | 2679 | 0.984 (0.974-0.994) | 0.001 | 0.018 |  |
| female | 3193 | 0.983 (0.974-0.991) | <0.001 | <0.018 |  |
| **Ethnic** |  |  |  |  | 0.986 |
| han | 5507 | 0.982 (0.975-0.989) | <0.001 | <0.018 |  |
| others | 365 | 0.981 (0.957-1.007) | 0.147 | 1.000 |  |
| **Education** |  |  |  |  | 0.195 |
| educated | 2483 | 0.979 (0.968-0.990) | <0.001 | <0.018 |  |
| no education | 3389 | 0.987 (0.979-0.995) | 0.002 | 0.036 |  |
| **Smoking** |  |  |  |  | 0.837 |
| no | 4648 | 0.984 (0.976-0.991) | <0.001 | <0.018 |  |
| yes | 1224 | 0.983 (0.968-0.997) | 0.018 | 0.324 |  |
| **Drinking** |  |  |  |  | 0.899 |
| no | 4658 | 0.983 (0.976-0.990) | <0.001 | <0.018 |  |
| yes | 1214 | 0.982 (0.968-0.997) | 0.019 | 0.342 |  |
| **Exercise** |  |  |  |  | 0.001 |
| no | 3983 | 0.990 (0.982-0.997) | 0.008 | 0.144 |  |
| yes | 1889 | 0.966 (0.955-0.978) | <0.001 | <0.018 |  |
| **Sleep.quality** |  |  |  |  | 0.675 |
| bad | 569 | 0.985 (0.964-1.006) | 0.151 | 1.000 |  |
| good | 3958 | 0.979 (0.971-0.987) | <0.001 | <0.018 |  |
| so so | 1345 | 0.988 (0.974-1.001) | 0.074 | 1.000 |  |

Adjusted for age, sex, ethnic, marital, education, smoking, drinking, income, exercise, occupation, sleep.hour, and sleep.quality.

# **Supplementary Table 5. Subgroup analysis between uPDI and all-cause mortality in CKM 0-2.**

| Subgroup | N | Adjusted HR (95% CI) | P value | Bonferroni-adjusted P value | P for interaction |
| --- | --- | --- | --- | --- | --- |
| Overall | 5872 | 1.012 (1.006-1.019) | <0.001 |  |  |
| Age |  |  |  |  | 0.176 |
| ˂75 | 1756 | 1.012 (0.994-1.031) | 0.202 | 1.000 |  |
| ˃84 | 2701 | 1.003 (0.996-1.010) | 0.401 | 1.000 |  |
| 75 to 84 | 1415 | 1.016 (1.003-1.030) | 0.016 | 0.288 |  |
| Sex |  |  |  |  | 0.474 |
| male | 2679 | 1.017 (1.008-1.026) | <0.001 | <0.018 |  |
| female | 3193 | 1.021 (1.013-1.029) | <0.001 | <0.018 |  |
| Ethnic |  |  |  |  | 0.346 |
| han | 5507 | 1.020 (1.013-1.026) | <0.001 | <0.018 |  |
| others | 365 | 1.034 (1.006-1.062) | 0.017 | 0.306 |  |
| Education |  |  |  |  | 0.120 |
| educated | 2483 | 1.019 (1.009-1.029) | <0.001 | <0.018 |  |
| no education | 3389 | 1.009 (1.001-1.017) | 0.026 | 0.468 |  |
| Smoking |  |  |  |  | 0.313 |
| no | 4648 | 1.018 (1.011-1.025) | <0.001 | <0.018 |  |
| yes | 1224 | 1.026 (1.012-1.041) | <0.001 | <0.018 |  |
| Drinking |  |  |  |  | 0.200 |
| no | 4658 | 1.018 (1.011-1.024) | <0.001 | <0.018 |  |
| yes | 1214 | 1.028 (1.014-1.043) | <0.001 | <0.018 |  |
| Exercise |  |  |  |  | 0.153 |
| no | 3983 | 1.014 (1.006-1.021) | <0.001 | <0.018 |  |
| yes | 1889 | 1.023 (1.012-1.034) | <0.001 | <0.018 |  |
| Sleep.quality |  |  |  |  | 0.203 |
| bad | 569 | 1.032 (1.011-1.053) | 0.002 | 0.036 |  |
| good | 3958 | 1.025 (1.017-1.032) | <0.001 | <0.018 |  |
| so so | 1345 | 1.009 (0.996-1.023) | 0.160 | 1.000 |  |

Adjusted for age, sex, ethnic, marital, education, smoking, drinking, income, exercise, occupation, sleep.hour, and sleep.quality.

# **Supplementary Table 6. Associations of plant-based diets with all-cause mortality by excluding deaths within the first three years of follow-up.**

| **Characteristic** |  |  | **Model 1** | | | **Model 2** | | |
| --- | --- | --- | --- | --- | --- | --- | --- | --- |
|  | **N** | **Event N** | **HR** | **95% CI** | ***p-value*** | **HR** | **95% CI** | ***p-value*** |
| **hPDI (continuous)** | 5,756 | 2,754 | 0.983 | 0.976, 0.990 | <0.001 | 0.987 | 0.981, 0.994 | <0.001 |
| **hPDI** |  |  |  |  |  |  |  |  |
| Q1 [26,42) | 1,258 | 676 | — | — |  | — | — |  |
| Q2 [42,46) | 1,410 | 662 | 0.870 | 0.781, 0.968 | 0.011 | 0.895 | 0.804, 0.997 | 0.044 |
| Q3 [46,49) | 1,282 | 615 | 0.899 | 0.805, 1.003 | 0.056 | 0.972 | 0.850, 1.060 | 0.354 |
| Q4 [49,71] | 1,806 | 801 | 0.794 | 0.716, 0.880 | <0.001 | 0.868 | 0.761, 0.936 | 0.001 |
| P for trend |  |  |  |  | <0.001 |  |  | 0.004 |
| **uPDI (continuous)** | 5,756 | 2,754 | 1.020 | 1.014, 1.026 | <0.001 | 1.014 | 1.008, 1.021 | <0.001 |
| **uPDI** |  |  |  |  |  |  |  |  |
| Q1 [25,46) | 1,287 | 498 | — | — |  | — | — |  |
| Q2 [46,50) | 1,279 | 611 | 1.205 | 1.071, 1.357 | 0.002 | 1.154 | 1.025, 1.300 | 0.018 |
| Q3 [50,55) | 1,694 | 816 | 1.200 | 1.073, 1.342 | 0.001 | 1.146 | 1.025, 1.282 | 0.017 |
| Q4 [55,71] | 1,496 | 829 | 1.433 | 1.282, 1.601 | <0.001 | 1.303 | 1.165, 1.458 | <0.001 |
| P for trend |  |  |  |  | <0.001 |  |  | <0.001 |
| Abbreviations: CI = Confidence Interval, HR = Hazard Ratio | | | | | | | | |
| Model 1 : no covariates were adjusted | | | | | | | | |
| Model 2 : adjusted for age, sex, ethnic, marital, education, smoking, drinking, income, exercise, occupation, sleep.hour, and sleep.quality | | | | | | | | |

# **Supplementary Table 7. To explore the association between a plant-based diet and mortality in CKM 0-2 stage (excluding participants who were classified as stage 2 due to dyslipidemia or chronic nephritis)**

| **Characteristic** |  |  | **Model 1** | | | **Model 2** | | |
| --- | --- | --- | --- | --- | --- | --- | --- | --- |
|  | **N** | **Event N** | **HR** | **95% CI** | ***p-value*** | **HR** | **95% CI** | ***p-value*** |
| **hPDI (continuous)** | 5,797 | 2,833 | 0.982 | 0.976, 0.989 | <0.001 | 0.987 | 0.980, 0.993 | <0.001 |
| **hPDI** |  |  |  |  |  |  |  |  |
| Q1 [26,42) | 1,270 | 699 | — | — |  | — | — |  |
| Q2 [42,46) | 1,422 | 683 | 0.865 | 0.778, 0.961 | 0.007 | 0.892 | 0.802, 0.992 | 0.035 |
| Q3 [46,49) | 1,294 | 632 | 0.886 | 0.796, 0.987 | 0.028 | 0.935 | 0.839, 1.042 | 0.225 |
| Q4 [49,71] | 1,811 | 819 | 0.786 | 0.710, 0.869 | <0.001 | 0.835 | 0.754, 0.925 | <0.001 |
| P for trend |  |  |  |  | <0.001 |  |  | 0.002 |
| **uPDI (continuous)** | 5,797 | 2,833 | 1.020 | 1.014, 1.026 | <0.001 | 1.010 | 1.004, 1.017 | 0.001 |
| **uPDI** |  |  |  |  |  |  |  |  |
| Q1 [25,46) | 1,291 | 511 | — | — |  | — | — |  |
| Q2 [46,50) | 1,289 | 631 | 1.218 | 1.084, 1.369 | <0.001 | 1.125 | 1.000, 1.265 | 0.050 |
| Q3 [50,55) | 1,712 | 844 | 1.216 | 1.089, 1.357 | <0.001 | 1.108 | 0.991, 1.238 | 0.071 |
| Q4 [55,71] | 1,505 | 847 | 1.438 | 1.288, 1.605 | <0.001 | 1.225 | 1.095, 1.370 | <0.001 |
| P for trend |  |  |  |  | <0.001 |  |  | 0.001 |
| Abbreviations: CI = Confidence Interval, HR = Hazard Ratio | | | | | | | | |
| Model 1 : no covariates were adjusted | | | | | | | | |
| Model 2 : adjusted for age, sex, ethnic, marital, education, smoking, drinking, income, exercise, occupation, sleep.hour, and sleep.quality | | | | | | | | |

# **Supplementary Table 8. Exclusion of baseline cancer participants to investigate the association between plant-based diet and all-cause mortality in CKM 0-2 stage.**

| **Characteristic** |  |  | **Model 1** | | | **Model 2** | | |
| --- | --- | --- | --- | --- | --- | --- | --- | --- |
|  | **N** | **Event N** | **HR** | **95% CI** | ***p-value*** | **HR** | **95% CI** | ***p-value*** |
| **hPDI (continuous)** | 5,825 | 2,843 | 0.982 | 0.976, 0.988 | <0.001 | 0.987 | 0.981, 0.994 | <0.001 |
| **hPDI** |  |  |  |  |  |  |  |  |
| Q1 [26,42) | 1,279 | 702 | — | — |  | — | — |  |
| Q2 [42,46) | 1,423 | 682 | 0.867 | 0.780, 0.963 | 0.008 | 0.895 | 0.805, 0.996 | 0.041 |
| Q3 [46,49) | 1,299 | 635 | 0.892 | 0.801, 0.993 | 0.037 | 0.949 | 0.851, 1.058 | 0.346 |
| Q4 [49,71] | 1,824 | 824 | 0.785 | 0.710, 0.868 | <0.001 | 0.851 | 0.768, 0.943 | 0.002 |
| P for trend |  |  |  |  | <0.001 |  |  | 0.009 |
| **uPDI (continuous)** | 5,825 | 2,843 | 1.020 | 1.014, 1.027 | <0.001 | 1.013 | 1.006, 1.019 | <0.001 |
| **uPDI** |  |  |  |  |  |  |  |  |
| Q1 [25,46) | 1,291 | 508 | — | — |  | — | — |  |
| Q2 [46,50) | 1,297 | 633 | 1.221 | 1.086, 1.372 | <0.001 | 1.128 | 1.001, 1.272 | 0.048 |
| Q3 [50,55) | 1,713 | 843 | 1.217 | 1.090, 1.358 | <0.001 | 1.133 | 1.009, 1.271 | 0.035 |
| Q4 [55,71] | 1,524 | 859 | 1.447 | 1.297, 1.615 | <0.001 | 1.266 | 1.126, 1.424 | <0.001 |
| P for trend |  |  |  |  | <0.001 |  |  | <0.001 |
| Abbreviations: CI = Confidence Interval, HR = Hazard Ratio | | | | | | | | |
| Model 1 : no covariates were adjusted | | | | | | | | |
| Model 2 : adjusted for age, sex, ethnic, marital, education, smoking, drinking, income, exercise, occupation, sleep.hour, and sleep.quality | | | | | | | | |

# **Supplementary Table 9. Exploring the association between plant-based diets and all-cause mortality in CKM 0=2 stages after excluding strict vegetarians.**

| **Characteristic** |  |  | **Model 1** | | | **Model 2** | | |
| --- | --- | --- | --- | --- | --- | --- | --- | --- |
|  | **N** | **Event N** | **HR** | **95% CI** | ***p-value*** | **HR** | **95% CI** | ***p-value*** |
| **hPDI (continuous)** | 5,793 | 2,826 | 0.982 | 0.975, 0.988 | <0.001 | 0.986 | 0.979, 0.993 | <0.001 |
| **hPDI** |  |  |  |  |  |  |  |  |
| Q1 [26,42) | 1,284 | 706 | — | — |  | — | — |  |
| Q2 [42,46) | 1,433 | 685 | 0.864 | 0.778, 0.960 | 0.006 | 0.888 | 0.799, 0.988 | 0.029 |
| Q3 [46,49) | 1,299 | 637 | 0.894 | 0.803, 0.996 | 0.042 | 0.947 | 0.849, 1.055 | 0.320 |
| Q4 [49,71] | 1,777 | 798 | 0.779 | 0.704, 0.862 | <0.001 | 0.834 | 0.752, 0.925 | <0.001 |
| P for trend |  |  |  |  | <0.001 |  |  | 0.003 |
| **uPDI (continuous)** | 5,793 | 2,826 | 1.020 | 1.014, 1.026 | <0.001 | 1.012 | 1.006, 1.019 | <0.001 |
| **uPDI** |  |  |  |  |  |  |  |  |
| Q1 [25,46) | 1,304 | 515 | — | — |  | — | — |  |
| Q2 [46,50) | 1,306 | 638 | 1.217 | 1.083, 1.367 | <0.001 | 1.125 | 0.998, 1.267 | 0.053 |
| Q3 [50,55) | 1,728 | 853 | 1.217 | 1.091, 1.358 | <0.001 | 1.135 | 1.012, 1.273 | 0.030 |
| Q4 [55,71] | 1,455 | 820 | 1.430 | 1.280, 1.596 | <0.001 | 1.255 | 1.115, 1.412 | <0.001 |
| P for trend |  |  |  |  | <0.001 |  |  | <0.001 |
| Abbreviations: CI = Confidence Interval, HR = Hazard Ratio | | | | | | | | |
| Model 1 : no covariates were adjusted | | | | | | | | |
| Model 2 : adjusted for age, sex, ethnic, marital, education, smoking, drinking, income, exercise, occupation, sleep.hour, and sleep.quality | | | | | | | | |

# **Supplementary Table 10. Construct a new Cox risk model (model 3) by incorporating chronic diseases as confounding factors and conduct sensitivity analysis.**

| **Characteristic** |  |  | **Model 1** | | | **Model 2** | | | **Model 3** | | |
| --- | --- | --- | --- | --- | --- | --- | --- | --- | --- | --- | --- |
|  | **N** | **Event N** | **HR** | **95% CI** | ***p-value*** | **HR** | **95% CI** | ***p-value*** | **HR** | **95% CI** | ***p-value*** |
| **hPDI (continuous)** | 5,872 | 2,870 | 0.982 | 0.976, 0.989 | <0.001 | 0.989 | 0.982, 0.995 | <0.001 | 0.989 | 0.983, 0.996 | 0.001 |
| **hPDI** |  |  |  |  |  |  |  |  |  |  |  |
| Q1 [26,42) | 1,289 | 707 | — | — |  | — | — |  | — | — |  |
| Q2 [42,46) | 1,438 | 690 | 0.867 | 0.781, 0.963 | 0.008 | 0.917 | 0.825, 1.020 | 0.111 | 0.914 | 0.821, 1.016 | 0.095 |
| Q3 [46,49) | 1,309 | 642 | 0.896 | 0.805, 0.998 | 0.045 | 0.972 | 0.872, 1.083 | 0.607 | 0.976 | 0.875, 1.087 | 0.655 |
| Q4 [49,71] | 1,836 | 831 | 0.789 | 0.713, 0.872 | <0.001 | 0.868 | 0.783, 0.961 | 0.007 | 0.867 | 0.782, 0.961 | 0.007 |
| P for trend |  |  |  |  | <0.001 |  |  | 0.02 |  |  | 0.023 |
| **uPDI (continuous)** | 5,872 | 2,870 | 1.020 | 1.014, 1.026 | <0.001 | 1.012 | 1.006, 1.019 | <0.001 | 1.009 | 1.002, 1.015 | 0.011 |
| **uPDI** |  |  |  |  |  |  |  |  |  |  |  |
| Q1 [25,46) | 1,304 | 515 | — | — |  | — | — |  | — | — |  |
| Q2 [46,50) | 1,306 | 638 | 1.217 | 1.083, 1.367 | <0.001 | 1.103 | 0.979, 1.242 | 0.107 | 1.102 | 0.978, 1.242 | 0.110 |
| Q3 [50,55) | 1,733 | 855 | 1.216 | 1.090, 1.356 | <0.001 | 1.097 | 0.978, 1.230 | 0.115 | 1.089 | 0.971, 1.222 | 0.145 |
| Q4 [55,71] | 1,529 | 862 | 1.438 | 1.289, 1.604 | <0.001 | 1.192 | 1.060, 1.340 | 0.003 | 1.186 | 1.054, 1.334 | 0.004 |
| P for trend |  |  |  |  | <0.001 |  |  | 0.006 |  |  | 0.009 |
| Abbreviations: CI = Confidence Interval, HR = Hazard Ratio | | | | | | | | |  |  |  |
| Model 1 : no covariates were adjusted | | | | | | | | |  |  |  |
| Model 2 : adjusted for age, sex, ethnic, marital, education, smoking, drinking, income, exercise, occupation, sleep.hour, and sleep.quality | | | | | | | | | | | |
| Model 3 : adjusted for age, sex, ethnic, marital, education, smoking, drinking, income, exercise, occupation, BMI, sleep.hour, sleep.quality, hypertension, diabetes, dyslipidemia and chronic nephritis. | | | | | | | | | | | |

# **Supplementary Table 11. Sensitivity analyses stratified by age, sex, CKM stage, and regular exercise.**

|  | Quartile 1 | Quartile 2 | Quartile 3 | Quartile 4 | Each 1-point increase |
| --- | --- | --- | --- | --- | --- |
| **hPDI** |  |  |  |  |  |
| **Age** |  |  |  |  |  |
| <75 | 1.00 (reference) | 0.860 (0.598-1.238) | 1.170 (0.827-1.655) | 0.895 (0.645-1.242) | 0.994 (0.974-1.014) |
| 75 to 84 | 1.00 (reference) | 1.019 (0.796-1.305) | 1.138 (0.888-1.459) | 0.949 (0.752-1.197) | 1.001 (0.992-1.009) |
| > 84 | 1.00 (reference) | 0.955 (0.842-1.082) | 0.999 (0.877-1.138) | 1.050 (0.928-1.188) | 0.995 (0.980-1.010) |
| **Sex** |  |  |  |  |  |
| male | 1.00 (reference) | 0.973 (0.822-1.152) | 1.024 (0.866-1.212) | 0.875 (0.748-1.023) | 0.985 (0.975-0.995) |
| female | 1.00 (reference) | 0.854 (0.744-0.981) | 0.938 (0.813-1.083) | 0.869 (0.758-0.997) | 0.988 (0.979-0.997) |
| **CKM** |  |  |  |  |  |
| stage 0 | 1.00 (reference) | 0.888 (0.782-1.008) | 1.005 (0.882-1.144) | 0.908 (0.803-1.027) | 0.993 (0.985-1.001) |
| stage 1 | 1.00 (reference) | 0.614 (0.430-0.877) | 0.554 (0.380-0.806) | 0.600 (0.432-0.835) | 0.965 (0.942-0.988) |
| stage 2 | 1.00 (reference) | 0.972 (0.766-1.234) | 0.976 (0.766-1.242) | 0.804 (0.639-1.012) | 0.982 (0.967-0.996) |
| **Exercise** |  |  |  |  |  |
| yes | 1.00 (reference) | 0.872 (0.713-1.067) | 0.828 (0.670-1.022) | 0.721 (0.590-0.880) | 0.968 (0.956-0.980) |
| no | 1.00 (reference) | 0.931 (0.821-1.055) | 1.021 (0.900-1.159) | 0.922 (0.818-1.040) | 0.993 (0.986-1.001) |
| **uPDI** |  |  |  |  |  |
| **Age** |  |  |  |  |  |
| <75 | 1.00 (reference) | 1.233 (0.885-1.716) | 1.063 (0.759-1.491) | 1.355 (0.962-1.909) | 1.015 (0.996-1.034) |
| 75 to 84 | 1.00 (reference) | 1.301 (1.001-1.691) | 1.185 (0.921-1.525) | 1.383 (1.070-1.788) | 1.003 (0.996-1.011) |
| > 84 | 1.00 (reference) | 1.001 (0.864-1.160) | 1.048 (0.911-1.206) | 1.063 (0.921-1.228) | 1.018 (1.004-1.031) |
| **Sex** |  |  |  |  |  |
| male | 1.00 (reference) | 1.010 (0.852-1.197) | 1.147 (0.974-1.350) | 1.303 (1.099-1.546) | 1.016 (1.007-1.025) |
| female | 1.00 (reference) | 1.244 (1.049-1.475) | 1.138 (0.965-1.342) | 1.251 (1.062-1.474) | 1.022 (1.013-1.030) |
| **CKM** |  |  |  |  |  |
| stage 0 | 1.00 (reference) | 1.042 (0.899-1.209) | 1.078 (0.938-1.240) | 1.156 (1.003-1.333) | 1.011 (1.003-1.019) |
| stage 1 | 1.00 (reference) | 1.445 (1.013-2.059) | 1.313 (0.917-1.880) | 1.602 (1.107-2.319) | 1.037 (1.018-1.057) |
| stage 2 | 1.00 (reference) | 1.234 (0.958-1.591) | 1.134 (0.881-1.460) | 1.371 (1.060-1.775) | 1.020 (1.008-1.033) |
| **Exercise** |  |  |  |  |  |
| yes | 1.00 (reference) | 1.211 (0.985-1.489) | 1.351 (1.103-1.655) | 1.336 (1.077-1.658) | 1.023 (1.012-1.034) |
| no | 1.00 (reference) | 1.081 (0.934-1.252) | 1.056 (0.918-1.214) | 1.212 (1.053-1.395) | 1.013 (1.005-1.020) |

Adjusted for age, sex, ethnic, marital, education, smoking, drinking, income, exercise, occupation, sleep.hour, and sleep.quality.

# **Supplementary Table 12. Baseline characteristics of participants excluded for missing diagnostic data versus the analytical sample.**

| **Characteristic** | **Excluded (N = 9,295)** | **Included (N = 5,872)** | **P value** |
| --- | --- | --- | --- |
| age, Median (Q1, Q3) | 92 (85, 100) | 83 (74, 91) | <0.001 |
| BMI, Median (Q1, Q3) | 19 (18, 22) | 20 (18, 23) | <0.001 |
| income, Median (Q1, Q3) | 13000 (5000, 30000) | 10000 (4000, 25000) | <0.001 |
| sleep.hour, Median (Q1, Q3) | 8 (6, 10) | 8 (6, 10) | <0.001 |
| hpdi, Median (Q1, Q3) | 44 (40, 47) | 46 (42, 49) | <0.001 |
| updi, Median (Q1, Q3) | 53 (48, 58) | 50 (46, 55) | <0.001 |
| **Classified variable** |  |  |  |
| Female, n (%) | 5587 (60.1%) | 3193 (54.4%) | <0.001 |
| Han, n (%) | 8711 (93.7%) | 5507 (93.8%) | 0.895 |
| No education, n (%) | 6313 (67.9%) | 3389 (57.7%) | <0.001 |
| Smoking, n (%) | 1365 (14.7%) | 1224 (20.8%) | <0.001 |
| Drinking, n (%) | 1370 (14.7%) | 1214 (20.7%) | <0.001 |
| Regular exercise, n (%) | 2141 (23.0%) | 1889 (32.2%) | <0.001 |
| Sleep quality: very good, n (%) | 1125 (12.1%) | 831 (14.2%) | <0.001 |
| Sleep quality: good, n (%) | 4720 (50.8%) | 3127 (53.3%) | <0.001 |
| Sleep quality: so so, n (%) | 2575 (27.7%) | 1345 (22.9%) | <0.001 |
| Sleep quality: bad, n (%) | 804 (8.6%) | 523 (8.9%) | <0.001 |
| Sleep quality: very bad, n (%) | 71 (0.8%) | 46 (0.8%) | <0.001 |
| Occupation: agriculture,forestry,animal husbandry or…, n (%) | 6168 (66.4%) | 4160 (70.8%) | <0.001 |
| Occupation: commercial, service or industrial worker…, n (%) | 1153 (12.4%) | 672 (11.4%) | <0.001 |
| Occupation: houseworker…, n (%) | 934 (10.0%) | 355 (6.0%) | <0.001 |
| Occupation: professional and technical personnel…, n (%) | 328 (3.5%) | 230 (3.9%) | <0.001 |

Continuous variables are presented as median (Q1, Q3) and were compared using the Kruskal-Wallis test; categorical variables are presented as n (%) and were compared using the Pearson chi-squared test. Excluded participants are those excluded because of missing baseline diagnostic data (hypertension, diabetes, heart disease, coronary heart disease/stroke, dyslipidaemia, or chronic nephritis).

# **Supplementary Table 13. Association between the overall plant-based diet index (PDI) and all-cause mortality in older adults with CKM stages 0-2 .**

| **PDI** | **Model** | **N** | **Events** | **HR (95% CI)** | **P value** |
| --- | --- | --- | --- | --- | --- |
| PDI (continuous, per 1-point increase) | Model 1 | 5872 | 2870.0 | 0.982 (0.976-0.988) | <0.001 |
| Q1 [27,46) | Model 1 | 1293 | 721.0 | 1.000 (reference) |  |
| Q2 [46,50) | Model 1 | 1279 | 626.0 | 0.908 (0.816-1.011) | 0.077 |
| Q3 [50,54) | Model 1 | 1540 | 758.0 | 0.897 (0.810-0.994) | 0.037 |
| Q4 [54,70] | Model 1 | 1759 | 764.0 | 0.749 (0.677-0.830) | <0.001 |
| P for trend | Model 1 |  |  | 0.980 (0.973-0.987) | <0.001 |
| PDI (continuous, per 1-point increase) | Model 2 | 5872 | 2870.0 | 1.001 (0.994-1.007) | 0.798 |
| Q1 [27,46) | Model 2 | 1293 | 721 | 1.000 (reference) |  |
| Q2 [46,50) | Model 2 | 1279 | 626 | 0.970 (0.870-1.082) | 0.582 |
| Q3 [50,54) | Model 2 | 1540 | 758 | 1.006 (0.905-1.120) | 0.907 |
| Q4 [54,70] | Model 2 | 1759 | 764 | 1.067 (0.956-1.191) | 0.245 |
| P for trend | Model 2 |  |  | 1.002 (0.995-1.010) | 0.584 |

Model 1: unadjusted. Model 2: adjusted for age, sex, ethnic, marital, education, smoking, drinking, income, exercise, occupation, sleep.hour, and sleep.quality. Quartile cut points were 46, 50, and 54. The proportional hazards assumption held globally (Schoenfeld test, P = 0.737).

# **Supplementary Table 13b. Association between PDI (continuous, per 1-point increase) and all-cause mortality by CKM stage.**

| **Model** | **CKM stage** | **N** | **Events** | **HR (95% CI)** | **P value** |
| --- | --- | --- | --- | --- | --- |
| Model 1 | stage 0 | 3736 | 1962 | 0.986 (0.979-0.992) | <0.001 |
| Model 1 | stage 1 | 811 | 289 | 0.958 (0.937-0.979) | <0.001 |
| Model 1 | stage 2 | 1325 | 619 | 0.991 (0.977-1.005) | 0.190 |
| Model 1 | stage P-interaction |  |  | 1.000 (reference) |  |
| Model 2 | stage 0 | 3736 | 1962 | 1.000 (0.992-1.007) | 0.921 |
| Model 2 | stage 1 | 811 | 289 | 0.990 (0.968-1.013) | 0.403 |
| Model 2 | stage 2 | 1325 | 619 | 1.009 (0.994-1.024) | 0.226 |
| Model 2 | stage P-interaction |  |  | 1.000 (reference) |  |

P for interaction (multiplicative): Model 1, P = 0.926; Model 2, P = 0.242. Model 2 adjusted as in Supplementary Table 13.

# **Supplementary Table 14. Sensitivity analyses for the association between PDI and all-cause mortality in CKM stages 0-2 (new in this revision).**

| **Sensitivity analysis** | **Model** | **Form** | **N** | **Events** | **HR (95% CI)** | **P value** |
| --- | --- | --- | --- | --- | --- | --- |
| S6 Exclusion of deaths occurring within the preceding 3 years of follow-up | Model 1 | continuous | 5756 | 2754 | 0.982 (0.976-0.988) | <0.001 |
| S6 Exclusion of deaths occurring within the preceding 3 years of follow-up | Model 1 | Q4 vs Q1 | 5756 | 2754 | 0.753 (0.679-0.836) | <0.001 |
| S6 Exclusion of deaths occurring within the preceding 3 years of follow-up | Model 2 | continuous | 5756 | 2754 | 1.001 (0.995-1.008) | 0.713 |
| S6 Exclusion of deaths occurring within the preceding 3 years of follow-up | Model 2 | Q4 vs Q1 | 5756 | 2754 | 1.073 (0.959-1.201) | 0.217 |
| S7 Exclude cases defined solely by dyslipidemia or nephritis for Stage 2. | Model 1 | continuous | 5797 | 2833 | 0.982 (0.976-0.988) | <0.001 |
| S7 Exclude cases defined solely by dyslipidemia or nephritis for Stage 2. | Model 1 | Q4 vs Q1 | 5797 | 2833 | 0.744 (0.671-0.824) | <0.001 |
| S7 Exclude cases defined solely by dyslipidemia or nephritis for Stage 2. | Model 2 | continuous | 5797 | 2833 | 1.000 (0.994-1.007) | 0.954 |
| S7 Exclude cases defined solely by dyslipidemia or nephritis for Stage 2. | Model 2 | Q4 vs Q1 | 5797 | 2833 | 1.056 (0.945-1.179) | 0.338 |
| S8 Exclusion of baseline cancer | Model 1 | continuous | 5825 | 2843 | 0.981 (0.975-0.987) | <0.001 |
| S8 Exclusion of baseline cancer | Model 1 | Q4 vs Q1 | 5825 | 2843 | 0.745 (0.673-0.826) | <0.001 |
| S8 Exclusion of baseline cancer | Model 2 | continuous | 5825 | 2843 | 1.001 (0.994-1.007) | 0.867 |
| S8 Exclusion of baseline cancer | Model 2 | Q4 vs Q1 | 5825 | 2843 | 1.065 (0.954-1.190) | 0.264 |
| S9 Exclude strict vegetarians | Model 1 | continuous | 5793 | 2826 | 0.981 (0.975-0.987) | <0.001 |
| S9 Exclude strict vegetarians | Model 1 | Q4 vs Q1 | 5793 | 2826 | 0.744 (0.671-0.824) | <0.001 |
| S9 Exclude strict vegetarians | Model 2 | continuous | 5793 | 2826 | 1.000 (0.994-1.007) | 0.971 |
| S9 Exclude strict vegetarians | Model 2 | Q4 vs Q1 | 5793 | 2826 | 1.056 (0.945-1.180) | 0.336 |
| S10 Model 3 with chronic diseases | Model 1 | continuous | 5872 | 2870 | 0.982 (0.976-0.988) | <0.001 |
| S10 Model 3 with chronic diseases | Model 1 | Q4 vs Q1 | 5872 | 2870 | 0.749 (0.677-0.830) | <0.001 |
| S10 Model 3 with chronic diseases | Model 2 | continuous | 5872 | 2870 | 1.001 (0.994-1.007) | 0.798 |
| S10 Model 3 with chronic diseases | Model 2 | Q4 vs Q1 | 5872 | 2870 | 1.067 (0.956-1.191) | 0.245 |
| S10 Model 3 with chronic diseases | Model 3 | continuous | 5872 | 2870 | 1.001 (0.995-1.008) | 0.689 |
| S10 Model 3 with chronic diseases | Model 3 | Q4 vs Q1 | 5872 | 2870 | 1.063 (0.951-1.188) | 0.281 |

Quartile cut points were 46, 50, and 54 (Q4 vs Q1). Model 1: unadjusted. Model 2: adjusted for age, sex, ethnic, marital, education, smoking, drinking, income, exercise, occupation, sleep.hour, and sleep.quality. Model 3: Model 2 additionally adjusted for BMI, hypertension, diabetes, chronic nephritis, and dyslipidaemia ; this extended model was intended solely as a robustness check.

# **Supplementary Table 15. Subgroup analysis between PDI (continuous, per 1-point increase) and all-cause mortality in CKM stages 0-2 (new in this revision).**

| **Subgroup** | **Stratum** | **N** | **Events** | **HR (95% CI)** | **P value** | **P for interaction** |
| --- | --- | --- | --- | --- | --- | --- |
| Age | 75 to 84 | 1415 | 590 | 1.000 (0.985-1.014) | 0.953 | 0.114 |
| Age | <75 | 1756 | 315 | 0.991 (0.972-1.011) | 0.385 | 0.114 |
| Age | >84 | 2701 | 1965 | 1.003 (0.995-1.011) | 0.477 | 0.114 |
| Sex | female | 3193 | 1601 | 1.007 (0.999-1.016) | 0.094 | 0.008 |
| Sex | male | 2679 | 1269 | 0.993 (0.983-1.002) | 0.130 | 0.008 |
| Ethnic | han | 5507 | 2676 | 1.000 (0.993-1.006) | 0.935 | 0.034 |
| Ethnic | others | 365 | 194 | 1.012 (0.987-1.038) | 0.342 | 0.034 |
| Education | educated | 2483 | 982 | 0.990 (0.979-1.001) | 0.073 | 0.014 |
| Education | no education | 3389 | 1888 | 1.006 (0.998-1.014) | 0.140 | 0.014 |
| Smoking | no | 4648 | 2313 | 1.003 (0.996-1.010) | 0.394 | 0.106 |
| Smoking | yes | 1224 | 557 | 0.991 (0.976-1.006) | 0.220 | 0.106 |
| Drinking | no | 4658 | 2309 | 0.999 (0.992-1.007) | 0.868 | 0.516 |
| Drinking | yes | 1214 | 561 | 1.009 (0.994-1.025) | 0.230 | 0.516 |
| Exercise | no | 3983 | 2091 | 1.006 (0.999-1.014) | 0.103 | 0.004 |
| Exercise | yes | 1889 | 779 | 0.986 (0.973-0.998) | 0.026 | 0.004 |
| Sleep.quality | bad | 569 | 253 | 0.999 (0.977-1.022) | 0.957 | 0.681 |
| Sleep.quality | good | 3958 | 1981 | 1.000 (0.992-1.008) | 0.973 | 0.681 |
| Sleep.quality | so so | 1345 | 636 | 1.005 (0.992-1.018) | 0.474 | 0.681 |

Adjusted for age, sex, ethnic, marital, education, smoking, drinking, income, exercise, occupation, sleep.hour, and sleep.quality (the stratifying variable was omitted from the model within each stratum). The Bonferroni-corrected significance threshold for 18 subgroup strata was 0.0028 (0.05/18); no stratum remained significant after correction.

# **Supplementary Table 16. Inverse probability-of-selection-weighted (IPSW) analyses of the associations between dietary indices and all-cause mortality (new in this revision).**

| **Index** | **Model** | **Form** | **HR (95% CI)** | **P value** |
| --- | --- | --- | --- | --- |
| HPDI | Model 1 | continuous | 0.980 (0.974-0.986) | <0.001 |
| HPDI | Model 1 | Q4 vs Q1 | 0.769 (0.697-0.848) | <0.001 |
| HPDI | Model 2 | continuous | 0.996 (0.989-1.004) | 0.365 |
| HPDI | Model 2 | Q4 vs Q1 | 0.966 (0.857-1.089) | 0.575 |
| UPDI | Model 1 | continuous | 1.018 (1.012-1.024) | <0.001 |
| UPDI | Model 1 | Q4 vs Q1 | 1.396 (1.251-1.556) | <0.001 |
| UPDI | Model 2 | continuous | 1.005 (0.997-1.013) | 0.208 |
| UPDI | Model 2 | Q4 vs Q1 | 1.095 (0.956-1.254) | 0.189 |
| PDI | Model 1 | continuous | 0.981 (0.976-0.987) | <0.001 |
| PDI | Model 1 | Q4 vs Q1 | 0.736 (0.666-0.814) | <0.001 |
| PDI | Model 2 | continuous | 0.999 (0.992-1.006) | 0.824 |
| PDI | Model 2 | Q4 vs Q1 | 1.032 (0.919-1.159) | 0.590 |

A logistic regression model for inclusion in the analytic sample was fitted on the pooled sample of 9,295 excluded and 5,872 included participants using all shared baseline covariates (age, sex, ethnic, marital, education, smoking, drinking, income, exercise, occupation, BMI, sleep.hour, sleep.quality, hPDI, and uPDI). Stabilized weights (mean 0.98; range 0.39-5.75) were applied in Cox models with robust standard errors. Model 1: unadjusted; Model 2: adjusted for age, sex, ethnic, marital, education, smoking, drinking, income, exercise, occupation, sleep.hour, and sleep.quality. This analysis addresses the largest source of differential exclusion (missing baseline diagnostic data); the additional exclusions due to prevalent cardiovascular disease (n = 1,225) and missing follow-up (n = 166) could not be modelled because their data were not extracted.

# **Supplementary Table 17. Correlations among PDI, hPDI, and uPDI (new in this revision).**

| **Index pair** | **Pearson r** | **Spearman ρ** |
| --- | --- | --- |
| PDI–hPDI | 0.632 | 0.593 |
| PDI–uPDI | -0.110 | -0.109 |
| hPDI–uPDI | -0.184 | -0.152 |

Computed among the 5,872 participants of the analytic sample. The moderate correlation between PDI and hPDI, the weak negative correlations involving uPDI, and the weak negative correlation between hPDI and uPDI indicate that the three indices capture related but distinct dimensions of plant-based eating.

# **Supplementary Figure 1. Restricted cubic spline for the association between PDI and all-cause mortality (new in this revision).**

Four knots at the 5th, 35th, 65th, and 95th percentiles of PDI; reference = median PDI (50); adjusted as Model 2 in Supplementary Table 13. P-overall = 0.887; P-nonlinear = 0.749.

# **Supplementary Figure 2. Kaplan-Meier survival curves for all-cause mortality according to PDI quartiles, overall and by CKM stage (new in this revision).**

|   Overall |   CKM stage 0 |
| --- | --- |
|   CKM stage 1 |   CKM stage 2 |

Log-rank P values: overall, P < 0.001; CKM stage 0, P < 0.001; CKM stage 1, P = 0.003; CKM stage 2, P = 0.535.
