# Supplementary material for: The associations between healthful and unhealthful plant-based dietary patterns and all-cause mortality among older Chinese adults with data-adapted early-stage cardiovascular-kidney-metabolic syndrome (stages 0–2): a prospective cohort study from the CLHLS
Source: Front Nutr. 2026 Oct 16;13:1915953. doi: 10.3389/fnut.2026.1915953 (PMC13721378; doi:10.3389/fnut.2026.1915953)
Supplement: Supplementary file 2 [file Supplementary_file_2.doc]

STROBE Statement—Checklist of items that should be included in reports of ***cohort studies***

|  | Item No | Recommendation |  |
| --- | --- | --- | --- |
| **Title and abstract** | 1 | (*a*) Indicate the study’s design with a commonly used term in the title or the abstract | Title: "A Prospective Cohort Study"Abstract: "Prospective cohort study" |
|  |  | (*b*) Provide in the abstract an informative and balanced summary of what was done and what was found | Abstract: Aims, Methods, Results, Conclusion (all presented) |
| Introduction | | |  |
| Background/rationale | 2 | Explain the scientific background and rationale for the investigation being reported | Introduction: Paragraphs 1–2 (CKM burden, dietary patterns, evidence gap) |
| Objectives | 3 | State specific objectives, including any prespecified hypotheses | Introduction: Last paragraph – "This study aimed to examine the associations of hPDI and uPDI with all-cause mortality..." |
| Methods | | |  |
| Study design | 4 | Present key elements of study design early in the paper | Methods – Study design and population: "This prospective cohort study used data from the CLHLS..." |
| Setting | 5 | Describe the setting, locations, and relevant dates, including periods of recruitment, exposure, follow-up, and data collection | Methods: CLHLS (23 provinces of China); 2008 wave (baseline); follow-up through March 2019 |
| Participants | 6 | (*a*) Give the eligibility criteria, and the sources and methods of selection of participants. Describe methods of follow-up | Methods: Age ≥65 years, CKM stages 0–2, no prevalent CVD. CLHLS database. Follow-up via death certificates or relatives/residents committee. |
|  |  | (*b*) For matched studies, give matching criteria and number of exposed and unexposed | Not applicable (no matching) |
| Variables | 7 | Clearly define all outcomes, exposures, predictors, potential confounders, and effect modifiers. Give diagnostic criteria, if applicable | Methods: Outcome = all-cause mortality; Exposure = hPDI/uPDI; Covariates listed (age, sex, lifestyle, etc.); CKM staging criteria explicitly defined (0/1/2) |
| Data sources/ measurement | 8* | For each variable of interest, give sources of data and details of methods of assessment (measurement). Describe comparability of assessment methods if there is more than one group | Methods: Dietary intake via FFQ; Mortality via official death certificates/relatives; Covariates via face-to-face interviews and physical measurements. See Supplementary Table S1 for details. |
| Bias | 9 | Describe any efforts to address potential sources of bias | Methods & Sensitivity analyses: Exclusion of early deaths (within 3 years); exclusion of strict vegetarians; exclusion of baseline cancer; multiple imputation for missing data; additional covariate adjustment |
| Study size | 10 | Explain how the study size was arrived at | Methods & Sensitivity analyses: Exclusion of early deaths (within 3 years); exclusion of strict vegetarians; exclusion of baseline cancer; multiple imputation for missing data; additional covariate adjustment |
| Quantitative variables | 11 | Explain how quantitative variables were handled in the analyses. If applicable, describe which groupings were chosen and why | Methods: hPDI/uPDI analyzed as continuous variables and quartiles. Quartiles chosen to examine dose–response trends. |
| Statistical methods | 12 | (*a*) Describe all statistical methods, including those used to control for confounding | Methods – Statistical Analysis: Cox proportional hazards models (crude and multivariable-adjusted); restricted cubic splines for dose–response |
|  |  | (*b*) Describe any methods used to examine subgroups and interactions | Methods: Subgroup analyses by CKM stage, age, sex, etc.; multiplicative interaction terms with likelihood ratio tests |
|  |  | (*c*) Explain how missing data were addressed | Methods: Multiple imputation (m = 5) by chained equations for covariates with low missingness (see Supplementary Table S5) |
|  |  | (*d*) If applicable, explain how loss to follow-up was addressed | Not explicitly stated (but exclusion of missing follow-up described in Figure 1) |
|  |  | (*e*) Describe any sensitivity analyses | Methods – Sensitivity analyses: Exclusion of early deaths, exclusion of dyslipidaemia/chronic nephritis-based CKM2, exclusion of cancer, exclusion of strict vegetarians, extended covariate adjustment |
| Results | | |  |
| Participants | 13* | (a) Report numbers of individuals at each stage of study—eg numbers potentially eligible, examined for eligibility, confirmed eligible, included in the study, completing follow-up, and analysed | Results – Participant Selection & Figure 1: 16,954 → 16,558 → 7,263 → 7,097 → 5,872 analysed |
|  |  | (b) Give reasons for non-participation at each stage | Results & Figure 1: Exclusions due to age <65 years (n=396), missing diagnosis (n=9,295), missing follow-up (n=166), prevalent CVD (n=1,225) |
|  |  | (c) Consider use of a flow diagram | Figure 1 (flowchart provided) |
| Descriptive data | 14* | (a) Give characteristics of study participants (eg demographic, clinical, social) and information on exposures and potential confounders | Results – Table 1: Baseline characteristics across CKM stages 0–2 (age, BMI, sleep, lifestyle, hPDI, uPDI, etc.) |
|  |  | (b) Indicate number of participants with missing data for each variable of interest | Methods – Statistical Analysis: "Multiple imputation (m = 5)... applied to covariates with low missingness (see Table S5)" |
|  |  | (c) Summarise follow-up time (eg, average and total amount) | Results: Median follow-up 108 months; 2,870 deaths occurred |
| Outcome data | 15* | Report numbers of outcome events or summary measures over time | Results: 2,870 deaths during median 108 months of follow-up. HRs with 95% CIs reported in Table 2. |
| Main results | 16 | (*a*) Give unadjusted estimates and, if applicable, confounder-adjusted estimates and their precision (eg, 95% confidence interval). Make clear which confounders were adjusted for and why they were included | Results – Table 2: Model 1 (crude) and Model 2 (multivariable-adjusted). Covariates listed in Methods and Table 2 footnote. |
|  |  | (*b*) Report category boundaries when continuous variables were categorized | Results – Table 2: Quartile boundaries for hPDI and uPDI (implied but not explicitly listed; see Supplementary Table S1 for scoring) |
|  |  | (*c*) If relevant, consider translating estimates of relative risk into absolute risk for a meaningful time period | None |
| Other analyses | 17 | Report other analyses done—eg analyses of subgroups and interactions, and sensitivity analyses | Results – Subgroup analysis, Sensitivity analyses, Supplementary Tables 4–11 |
| Discussion | | |  |
| Key results | 18 | Summarise key results with reference to study objectives | Discussion – First paragraph: hPDI associated with lower mortality (HR=0.868 for highest quartile); uPDI associated with higher mortality (HR=1.192). More evident in CKM stages 1–2. |
| Limitations | 19 | Discuss limitations of the study, taking into account sources of potential bias or imprecision. Discuss both direction and magnitude of any potential bias | Discussion – Limitations paragraph (p. 17): Adapted CKM definition (no laboratory biomarkers); self-reported dietary intake; residual confounding; all-cause mortality only; generalisability to non-Chinese populations; observational design precludes causality; selection bias possible. |
| Interpretation | 20 | Give a cautious overall interpretation of results considering objectives, limitations, multiplicity of analyses, results from similar studies, and other relevant evidence | Discussion – Throughout: "The stronger associations observed in CKM stages 1–2 may be biologically plausible..." and "null or weaker associations in CKM stage 0 should not be interpreted as evidence that diet is unimportant" |
| Generalisability | 21 | Discuss the generalisability (external validity) of the study results | Discussion – Limitations & Conclusions: Findings may not be directly generalisable to younger populations or populations with different dietary structures and healthcare contexts. |
| Other information | | |  |
| Funding | 22 | Give the source of funding and the role of the funders for the present study and, if applicable, for the original study on which the present article is based | Funding section: National Natural Science Foundation of China (Grant No. 82474587) and Innovation Fund of Health Commission of Chengdu (No. WXLH202403138). Role of funders not explicitly stated. |

*Give information separately for exposed and unexposed groups.

**Note:** An Explanation and Elaboration article discusses each checklist item and gives methodological background and published examples of transparent reporting. The STROBE checklist is best used in conjunction with this article (freely available on the Web sites of PLoS Medicine at http://www.plosmedicine.org/, Annals of Internal Medicine at http://www.annals.org/, and Epidemiology at http://www.epidem.com/). Information on the STROBE Initiative is available at http://www.strobe-statement.org.
